# Region of interest-specific loss functions improve T_2_ quantification with ultrafast T_2_ mapping MRI sequences in knee, hip and lumbar spine

**DOI:** 10.1038/s41598-022-26266-z

**Published:** 2022-12-23

**Authors:** Aniket A. Tolpadi, Misung Han, Francesco Calivà, Valentina Pedoia, Sharmila Majumdar

**Affiliations:** Department of Radiology and Biomedical Imaging, University of California, 1700, 4th Street, San Francisco, CA 94158 USA

**Keywords:** Cartilage, Magnetic resonance imaging, Biomedical engineering

## Abstract

MRI T_2_ mapping sequences quantitatively assess tissue health and depict early degenerative changes in musculoskeletal (MSK) tissues like cartilage and intervertebral discs (IVDs) but require long acquisition times. In MSK imaging, small features in cartilage and IVDs are crucial for diagnoses and must be preserved when reconstructing accelerated data. To these ends, we propose region of interest-specific postprocessing of accelerated acquisitions: a recurrent UNet deep learning architecture that provides T_2_ maps in knee cartilage, hip cartilage, and lumbar spine IVDs from accelerated T_2_-prepared snapshot gradient-echo acquisitions, optimizing for cartilage and IVD performance with a multi-component loss function that most heavily penalizes errors in those regions. Quantification errors in knee and hip cartilage were under 10% and 9% from acceleration factors R = 2 through 10, respectively, with bias for both under 3 ms for most of R = 2 through 12. In IVDs, mean quantification errors were under 12% from R = 2 through 6. A Gray Level Co-Occurrence Matrix-based scheme showed knee and hip pipelines outperformed state-of-the-art models, retaining smooth textures for most R and sharper ones through moderate R. Our methodology yields robust T_2_ maps while offering new approaches for optimizing and evaluating reconstruction algorithms to facilitate better preservation of small, clinically relevant features.

## Introduction

Magnetic Resonance Imaging (MRI) has emerged as a crucial part of diagnosing pathologies such as osteoarthritis, ligament damage, tumors, and others^1–3^. Within MRI, several sequences can be deployed that exploit intrinsic tissue properties, providing images of varying weightings that effectively visualize tissues such as muscle, ligaments, bone marrow, and others^4^. In musculoskeletal (MSK) applications, clinical imaging protocols consist mostly of 2D fast spin echo (FSE) acquisitions with T_1_ or T_2_ weighting in various acquisition planes, which do well in depicting the structure and morphology of the underlying anatomy^5^. However, compositional MRI (cMRI) techniques to assess actual tissue parameters are gaining more attention as a complement of qualitative imaging.

cMRI techniques like T_2_ relaxometry can provide maps of T_2_ values (or another intrinsic MR parameter) across an imaging volume rather than a morphological image. For MSK applications, T_2_ relaxometry offers sensitivity to water content, collagen content, and collagen fiber orientation in cartilage^6^, making it sensitive to biochemical changes that can precede morphological changes across several tissues and anatomies^7,8^. Pre-morphological change sensitivity has been best characterized in the knee, where T_2_ values are significantly higher across most cartilage compartments in healthy patients that later develop osteoarthritis (OA) compared to controls^9,10^. Additionally, T_2_ relaxometry offers quantitative MSK tissue health assessments, correlating with measures of hip cartilage and intervertebral disc (IVD) health^11,14–16^, whereas in conventional clinical imaging, only semiquantitative tissue health assessments are obtainable with expert annotation^12,13^. All of this makes cMRI a promising potential addition to clinical imaging protocols.

A major challenge facing clinical adoption of cMRI, however, is acquisition time: while mapping sequences like the magnetization-prepared angle-modulated partitioned k-space spoiled gradient echo snapshots (MAPSS) can provide robust MR parameter maps, their acquisition times can exceed 5–6 minutes, making their addition to a clinical scan protocol difficult^17^. Acquisitions can be accelerated by sampling fewer points in k-space, inducing aliasing artifacts in resulting images that must be removed through subsequent postprocessing. Some proposed approaches to these ends are reconstruction strategies such as parallel imaging (PI), compressed sensing (CS), model-based reconstructions, deep learning (DL), low-rank and sparse modeling methods, and MR Fingerprinting (MRF). Most of these approaches design an algorithm or exploit the redundancy of k-space acquisition across multiple coils to predict the appearance of the fully-sampled reconstructed image.

PI was one of the earliest techniques to accelerate MRI acquisition and has seen clinical adoption. Here, the redundancy of a multiple coil acquisition is leveraged to mitigate aliasing artifacts^18–20^, reducing clinical scan time up to acceleration factor R = 3 for MSK applications^21,22^. CS^23^ has also shown promise, where aliased images are iteratively reconstructed by minimizing an objective function, retaining fidelity to acquired k-space and imposing sparsity on the reconstructed image in another domain. CS has attained clinically acceptable MSK image quality through roughly R = 4^21,22,24,25^, and up to R = 8 in research settings for knee cartilage T_1ρ_ mapping^26^. Similarly, PI and CS have also been applied sequentially (and simultaneously) for further acceleration^27^.

For cMRI acceleration, model-based reconstructions have gained traction, integrating the physics of T_2_/T_2_^*^ decay and T_1_ recovery into an objective function iteratively optimized to reconstruct maps, showing promise in brain and lumbar spine T_2_ mapping^28–30^. More generally, incorporation of the physics of MRI parameter recovery/decay has seen applications not just in model-based approaches, but in various aspects of other methodologies as well^31^. DL approaches have gained prominence in solving inverse problems such as reconstruction, allowing for cMRI reconstructions at higher R than other methods. Standalone DL approaches have seen promising results in knee MAPSS acceleration, T_1_ mapping, and T_2_ mapping sequences^32–36^. In other methodologies, DL has been integrated with model-based approaches while introducing loss functions to maintain fidelity to acquired k-space, seeing promise up to R = 8 in knee and brain T_1_ and T_2_ mapping^37–39^. DL has been applied to accelerate T_2_ mapping in MR Fingerprinting, where DL can remove aliasing artifacts from undersampled acquisitions and/or replacing time-consuming dictionary lookup steps to predict MR parameter maps, and exploiting spatial correlations within maps to improve reconstructions^40,41^. Lastly, aside from DL, low-rank and sparse modeling methods have emerged as a means of accelerating acquisitions, where several MRI images acquired at different echo times are decomposed into temporal basis functions and spatial coefficients to model an MRI parameter, showing promise through R = 8^42^.

These works represent great progress, although avenues for improvement remain. Above all, these methods have optimized reconstructed images for full-volume performance; however, in MSK applications, clinical assessment relies on the inspection of precise anatomic features in specific anatomic regions, and consequently, the reconstruction quality cannot be compromised within these regions. Put differently, given clinical context, strong image quality may be most important in specific regions of an image, leaving room for algorithm optimization. Furthermore, most recent published approaches leverage k-space data in formal reconstruction approaches, but for niche applications such as region of interest (ROI)-focused optimization, such approaches may be outperformed by DL-based post-processing algorithms that denoise and fit undersampled T_2_-weighted images without using raw k-space. Moreover, performance of standard reconstruction algorithms is typically evaluated using metrics such as structural similarity index (SSIM), normalized root mean square error (NRMSE), and peak signal-to-noise ratio (PSNR), but recent works show these metrics may not provide the best correspondence with radiologist annotations^43,44^, leading other groups to propose alternate metrics to fill this niche^45^.

To these ends, this study proposes a recurrent UNet pipeline to postprocess undersampled coil-combined T_2_-weighted echo images, fitting and predicting T_2_ maps from accelerated MAPSS acquisitions in the knee, hip and lumbar spine^46,47^. These algorithms are trained with multi-component, ROI-specific losses that optimize predicted maps for T_2_ value and textural retention in cartilage and IVDs. In doing so, our approach allows for ROI-specific optimization, facilitating retention of small, crucial clinical features in tissues of interest while building on past applications of weighted loss functions for image processing tasks^48^.

To summarize, the contributions of this work are as follows:

- By using a 4-component loss function in network training, we introduce the concept of “ROI-specific optimization” of cMRI accelerated acquisition pipelines.
- We conduct a thorough ablation study of these 4 loss function components, proving the value of all in retaining textures in predicted maps while retaining high fidelity to ground truth T_2_ values.
- Acknowledging that standard evaluation metrics such as SSIM and NRMSE provide suboptimal sensitivity to clinically relevant metrics, we conduct a thorough Gray Level Co-Occurrence Matrix (GLCM)-metric-based analysis of smooth and sharp textural retention in predicted maps, with an eye towards better evaluation of retention of small features crucial to clinical diagnoses^49,50^.
- We build on limited literature in hip and lumbar spine cMRI accelerated acquisition schemes by developing and evaluating our pipeline not only in knee cartilage, as several other works have done, but also for hip cartilage and lumbar spine IVD in ultrafast acquisitions.

## Methods

### MAPSS acquisitions

Retrospective datasets including MAPSS in the knee (n = 244 patients, 446 scans), hip (n = 67 patients, 89 scans), and lumbar spine (n = 21 patients, 24 scans) acquired from clinical 3 T MRI scanners was used. Patients were scanned in accordance with all pertinent guidelines, including approval from the University of California, San Francisco Institutional Review Board (Human Research Protection Program), and informed consent was obtained from all study participants. MAPSS simultaneously acquired multiple T_1ρ_ and T_2_ weighted images, using T_1ρ_ or T_2_ preparation followed by 3D RF-spoiled gradient-echo Cartesian acquisition in a segmented radial centric view ordering during a transient state. A fat-selective inversion pulse was applied before either T_1ρ_^51,52^ or T_2_ preparation^53^. Each acquisition included T_1ρ_-prepared images at four spin-lock times (TSLs) for T_1ρ_ quantification, and three additional T_2_-prepared images for T_2_ quantification (TSL = 0 ms images were shared for TE = 0 ms images). In this study, only T_2_-prepared images at four different TEs and corresponding T_2_ maps from the MAPSS sequence were used. k_y_-k_z_ space was acquired within an elliptical coverage (area = 0.7 compared to rectangular k_y_-k_z_, not acquiring corner space). Knee images were acquired from patients having ACL injuries, with scans taken at baseline and 3 years post-reconstruction. Hip images were acquired from patients having hip OA. Lumbar spine images were acquired from healthy subjects or patients with low back pain. Table 1 shows acquisition parameters.

**Table 1:** Knee, hip and lumbar spine datasets and splits.

		Knee	Hip	Lumbar spine
Acquisition parameters	Scanner (s)	GE Discovery MR750w (GE Healthcare, Waukesha, WI), GE Discovery MR750 (GE Healthcare, Waukesha, WI)	GE Discovery MR750w (GE Healthcare, Waukesha, WI), GE Discovery MR750 (GE Healthcare, Waukesha, WI)	GE Discovery MR750w (GE Healthcare, Waukesha, WI), GE Signa PET/MR (GE Healthcare, Waukesha, WI)
	Coil(s)	8-channel T/R knee array (Invivo, Gainesville, FL)	32-channel cardiac array (Invivo, Gainesville, FL)	Geometry embracing method (GEM) posterior array (GE Healthcare, Aurora, OH)
	FOV	14 × 14 cm^2^	14 × 14 cm^2^	20 × 20 cm^2^
	acquisition matrix	256 × 128	256 × 128	256 × 128
	Slice thickness	4.0 mm	4.0 mm	8.0 mm
	Slices	22	28	12
	TEs	0 ms, 12.9 ms, 25.7 ms, 51.4 ms	0 ms, 10.4 ms, 20.8 ms, 41.7 ms	0 ms, 12.9 ms, 25.7 ms, 51.4 ms
	Readout BW	± 62.5 kHz	± 62.5 kHz	± 62.5 kHz
	Magnetization Recovery time	1.3 s	1.2 s	1.5 s
	ARC	2X	2X	None
	no phase wrap	None	2X k_y_ oversampling	None
	Other	64-view acquisition/T_2_ preparation	64-view acquisition/T_2_ preparation	64-view acquisition/T_2_ preparation
Demographics information	Sex (M/F)	140/104	35/32	10/11
	Age	29.7 ± 12.9	48.9 ± 13.2	45.3 ± 14.7
	Weight	74.3 ± 12.7 kg	69.8 ± 12.4 kg	69.6 ± 11.0 kg
Training information details	Learning rate	0.001	0.001	0.001
	Batch size	1	1	1
	Number of batches used for training	R = 2: 40,390 R = 3: 36,351 R = 4: 36,351 R = 6: 40,390 R = 8: 60,585 R = 10: 36,351 R = 12: 40,390	R = 2: 39,030 R = 3: 28,622 R = 4: 26,020 R = 6: 39,030 R = 8: 36,428 R = 10: 26,020 R = 12: 39,030	R = 2: 1120 R = 3: 1344 R = 4: 1120 R = 6: 1120 R = 8: 2016 R = 10: 1344 R = 12: 3360
Training	Patients	144	39	13
	Scans	265	59	14
	Slices	5591	1533	112
Validation	Patients	50	15	4
	Scans	91	15	5
	Slices	1952	390	42
Test	Patients	50	13	4
	Scans	90	15	5
	Slices	1928	390	40
Total	Patients	244	67	21
	Scans	446	89	24
	Slices	9471	2313	194

MAPSS acquisition parameters for all datasets, with corresponding training, validation and test splits. ARC refers to Auto-calibrating Reconstruction for Cartesian Imaging^70^. For hip acquisitions, no phase wrap was applied: k_y_ was oversampled by a factor of 2X, with space outside the prescribed y-FOV eliminated after reconstruction. In some cases, multiple acquisitions were taken per patient due to having multiple knees/hips scanned, or due to having follow-up scans for the same patient. Age and weight are reported mean ± 1 standard deviation (s.d.). Datasets were split into training, validation and test, ensuring all scans of a particular patient were only placed into one of the three datasets. Unless otherwise noted, all results are reported on the test set are described by this table; to ensure robustness of trained pipeline to data splits, additional versions were trained on 2 more splits detailed in Supplementary Tables S2 and S13, with results on those splits described in Supplementary Tables S14, S15, S16.

### T_2_ Fitting and spatial undersampling

Later T_2_ weighted echo time images for each slice were registered to corresponding TE = 0 ms images using a 3D rigid registration algorithm with a normalized mutual information criterion^54^. Levenberg–Marquardt fitting of registered T_2_ weighted images yielded ground truth T_2_ maps^55^.

To simulate accelerated acquisition, coil-combined T_2_ weighted magnitude images after reconstruction (ARC for knee and hip) were Fourier transformed and retrospectively undersampled using a center-weighted Poisson disc pattern, fully sampling a central 5% square in k_y_-k_z_ (R = 2, 3, 4, 6, 8, 10, 12). Acquisition times associated with ground truth and accelerated MAPSS acquisitions in each body part can be found in Supplementary Table S1. As MAPSS acquires phase-encode lines with elliptical coverage in k_y_-k_z_ (relative area of 0.7 compared to rectangular coverage), phase encoding lines solely within the sampling ellipse were undersampled. Although working with synthesized k-space data generated from coil-combined magnitude images, retrospective undersampling was done and R reported with respect to elliptical coverage in k_y_-k_z_ to accurately simulate an actual undersampling pattern and not overstate model performance^56^. However, for hip acquisitions, reconstructed space outside the y-FOV had already been discarded; thus, simulating acquisitions with application of ‘no phase wrap’ was not possible and undersampling patterns would differ from those implemented on a scanner. T_2_ weighted images from each echo time were undersampled with a unique pattern. For k_y_-k_z_ lines not sampled at a given echo time, those k_y_-k_z_ lines were initialized with the corresponding k_y_-k_z_ from the image with the temporally closest echo time for which that k_y_-k_z_ was sampled. Only k_y_-k_z_ lines not sampled in images acquired at all echo times were zero-filled. k-Space was subsequently inverse Fourier transformed, yielding undersampled, aliased images.

### DL pipeline training

#### DL architecture

**Figure 1:**
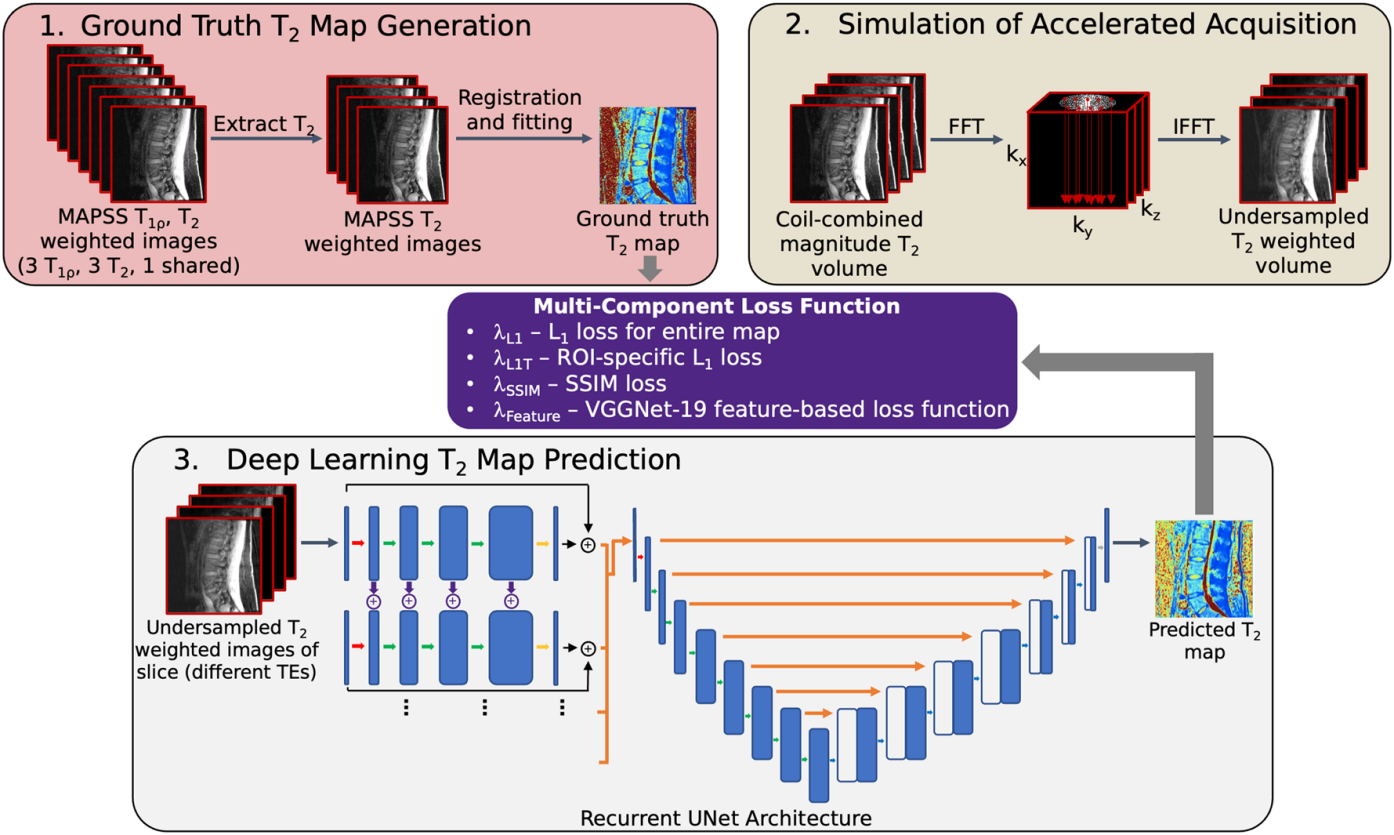
Proposed pipeline. Experiments in proposed study entail generating ground truth T_2_ maps from MAPSS, simulating accelerated acquisition of T_2_-weighted MAPSS images, and training a network to predict T_2_ maps from undersampled images. (1) MAPSS contains 7 images, 3 that are T_2_ weighted, 3 T_1ρ_ weighted, and 1 shared; the T_2_ and shared image weightings are extracted, registered, and fitted slice-wise to yield ground truth T_2_ maps. To simulate accelerated acquisition, each volume of coil-combined magnitude T_2_ weighted images acquired at a given echo time are Fourier transformed, undersampled along the k_y_–k_z_ plane with a center-weighted Poisson disc pattern, and inverse Fourier transformed to yield a simulated accelerated acquisition of a volume. Finally, undersampled T_2_ weighted images acquired at all echo times for the same anatomic slice are concatenated and fed to the proposed recurrent UNet architecture, which predicts the T_2_ map appearance for the slice. Training is done slice-wise with a multi-component loss function that includes a novel ROI-specific L_1_ loss that optimizes predicted T_2_ maps in cartilage and IVD ROIs, with other components that improve training stability and encourage retention of textures.

An overview of the data processing and training schemes is shown in Fig. 1, while a detailed diagram depicting our proposed network architecture is in Supplementary Fig. S1 (“Full Model”; 39,808,710 trainable parameters). Magnitude images from data undersampled as specified were fed into a recurrent UNet network. The network contains an initial recurrent portion: aliased images from each T_2_ echo time have a 5-layer processing stream of 2D 3 × 3 convolutions with stride 1, yielding layers of depth 64, 128, 256, 512, and 1. Residual connections connect input aliased images with processing stream outputs. 2D 3 × 3 convolutions with stride 1 and residual connections transfer information between temporally adjacent corresponding hidden echo time processing layers with weighting parameter λ_w_ = 0.2^57^. This soft-weighted view-sharing of neighboring T_2_ weighted echo time images facilitated sharing of feature map information between temporally adjacent echo time images, which can augment sharing of k_y_-k_z_ initializations to improve network image predictions. Outputs of all 4 echo time image processing streams were concatenated and fed to a UNet that predicted T_2_ maps. 2D 3 × 3 convolutions with stride 2 were used for the encoder, and 2D 4 × 4 transpose convolutions with stride 2 for the decoder. Two additional architecture versions were also trained: one UNet with no recurrent portion (“No RNN”; 35,116,037 trainable parameters) and a second in which all layers apart from inputs to the recurrent portion and UNet had half the depth listed in Supplementary Fig. S1 (“Reduced Parameters”; 9,958,246 trainable parameters).

#### Loss function

Networks were trained with the multi-part loss function shown in Eq. (1):

1 $$L_{network} = \lambda_{{L_{1} }} L_{{L_{1} }} + \lambda_{{L_{1,\phi } }} L_{{L_{1, \phi } }} + \lambda_{SSIM} L_{SSIM} + \lambda_{Feature} L_{Feature}$$

in which $L_{{L_{1} }}$ is a scaled global L_1_ loss detailed in Eq. (2):

2 $$L_{{L_{1} }} = \left| {S\left( {T_{2} } \right) - S\left( {\hat{T}_{2} } \right)} \right|$$

where $T_{2}$ represents ground truth T_2_, $\hat{T}_{2}$ represents predicted T_2_, and $S\left( x \right)$ is a translated and scaled sigmoid operator that assigns more weight to higher T_2_ values. Sharp contrasts and high $T_{2}$ values can easily be lost in accelerated acquisition schemes, so $S\left( x \right)$ proved useful through empirical testing in focusing networks to preserve these details. $S\left( x \right)$ is defined below in Eq. (3):

3 $$S\left( x \right) = y_{l} + \left( {y_{h} - y_{l} } \right)\left( {1 + exp\left( { - \left( {10/\left( {x_{h} - x_{l} } \right)} \right)\left( {x - \left( {x_{l} + x_{h} } \right)/2 } \right)} \right)} \right)^{ - 1}$$

where $x_{l}$, $x_{h}$ were the low and high T_2_ value limits where the sigmoid operator weighting will transition from $y_{l}$ to $y_{h}$. Parameters selected for the knee were as follows: $x_{l}$ = 0 ms, $x_{h}$ = 100 ms, $y_{l}$ = 0.1, $y_{h}$ = 1.0. In the hip: $x_{l}$ = 0 ms, $x_{h}$ = 60 ms, $y_{l}$ = 0.5, $y_{h}$ = 1.0. In the lumbar spine: $x_{l}$ = 0 ms, $x_{h}$ = 150 ms, $y_{l}$ = 0.25, $y_{h}$ = 1.0. A schematic of the operator that results from parameters of all three anatomies can be found as Supplementary Fig. S2.

$L_{{L_{1, \phi } }}$ is the ROI-specific L_1_ loss, and is described in Eq. (4):

4 $$L_{{L_{1, \phi } }} = \left| {S\left( {T_{2,\phi } } \right) - S\left( {\hat{T}_{2,\phi } } \right))} \right|$$

where $T_{2,\phi }$ were ground truth T_2_ values in the tissue of interest $\phi$ (IVD or cartilage), scaled by $S\left( x \right)$ (Eq. (3)), and $\hat{T}_{2,\phi }$ is the same for predicted T_2_. Pixels corresponding to $\phi$ are obtained from segmentation masks, the generation of which is described in “Training and Segmentation Details”. For both $L_{{L_{1} }}$ and $L_{{L_{1, \phi } }}$, L_1_ norms were used instead of L_2_ due to reduced sensitivity to outliers, leading to more stable trainings.

$L_{SSIM}$ is an SSIM loss, described in Eq. (5):

5 $$L_{SSIM} = 1 - SSIM$$

where SSIM was the structural similarity index between predicted and target maps.

$L_{Feature}$ is a feature-based loss function designed to retain sharper textures, calculated as in Eq. (6):

6 $$L_{Feature} = \left| {VGG_{{T_{2} }} - VGG_{{\hat{T}_{2} }} } \right|$$

where $VGG_{{T_{2} }}$ and $VGG_{{\hat{T}_{2} }}$ were the outputs of the 21st layer of a VGG-19^58^ network pretrained on ImageNet when fed resized and normalized target and predicted T_2_ maps, respectively. Maps were resized to 224 × 224 × 1, concatenated with themselves along the channel axis to yield 224 × 224 × 3 inputs, and normalized such that the channels had mean pixel values of 0.485, 0.456 and 0.406, with standard deviations of 0.229, 0.224, and 0.225, respectively.

$\lambda_{{L_{1} }}$,$\lambda_{{L_{1,\phi } }}$, $\lambda_{SSIM}$, $\lambda_{Feature}$ were loss component weightings. All were positive-valued and optimized through constrained random hyperparameter searches with the following ranges:

- Knee: $$\lambda_{{L_{1} }}$$ = 1,$$\lambda_{{L_{1,\phi } }} = 50 - 150$$, $$\lambda_{SSIM} = 0 - 2$$, $$\lambda_{Feature} = 0 - 0.5$$
- Hip: $$\lambda_{{L_{1} }}$$ = 1,$$\lambda_{{L_{1,\phi } }} = 0 - 3$$, $$\lambda_{SSIM} = 0 - 2$$, $$\lambda_{Feature} = 0 - 1$$.
- Spine: $$\lambda_{{L_{1} }}$$ = 1,$$\lambda_{{L_{1,\phi } }} = 1 - 10$$, $$\lambda_{SSIM} = 10 - 100$$, $$\lambda_{Feature} = 5 - 55$$.

#### Training and segmentation details

Scans of all three anatomies were split into training, validation and test sets as shown in Table 1. In the knee, cartilage was segmented manually. In the hip, cartilage was segmented manually for 4 central slices per volume. Segmentation in both was performed by research assistants trained by radiologists with over 20 years of experience. Since the hip dataset had substantially fewer segmented than unsegmented slices, the hip training set was bootstrapped to equalize the number of slices with and without segmentations (1068 bootstrapped slices). Finally, in the lumbar spine, IVDs were segmented with an ensemble of coarse-to-fine context memory (CFCM) networks^59^. To calculate performance metrics and implement ROI-specific training losses, these segmentation masks were leveraged to identify pixels in tissues of interest (cartilage or IVD).

Signal values were scaled per slice for the middle 95% of pixel values to fall between 0 and 500 for the knee and lumbar spine, and 0 and 100 for the hip; these ranges were optimized empirically. During training, imaging volumes were augmented with random translation (± 10 pixels across phase and frequency directions) and random rotation (± 5 degrees about slice direction). All models were trained with learning rate 0.001 and Adam optimizer on an NVIDIA Titan Xp 12 GB GPU with batch size of 1 so the model would fit on a single GPU. Separate pipelines were trained for all 3 anatomies at R = 2, 3, 4, 6, 8, 10, and 12. For each pipeline, and at each trained R, a constrained random hyperparameter search was done for 15 iterations at 10 epochs per iteration to optimize $\lambda_{{L_{1} }}$,$\lambda_{{L_{1,\phi } }}$, $\lambda_{SSIM}$, and $\lambda_{Feature}$ for visual fidelity of predicted maps to ground truth. Visual fidelity was assessed in the search using NRMSE (calculated as shown in Eq. (7)) and Pearson’s r in the tissue of interest^60^.

7 $$NRMSE={{\Vert {T}_{2}-\widehat{{T}_{2}}\Vert }_{2,\phi }\left({\Vert {T}_{2}\Vert }_{2,\phi }\right)}^{-1}$$

Final pipelines across all anatomies and R were trained using optimized parameter sets until validation loss did not decrease for 10 epochs. Key training details are summarized as part of Table 1.

## Experiments

### Loss function ablation study

An ablation study is key to understand contributions of loss components. Given optimized loss function weights, every combination of loss components was ablated and corresponding models were retrained until validation loss no longer decreased. “No RNN” and “Reduced Parameters” networks were also trained while maintaining loss function components at optimized values to assess the utility of simpler architectures. NRMSE and Pearson’s correlation coefficient (r) were calculated in tissues of interest across the test set for original and ablated models to determine loss component contributions to performance. Pearson’s r was deemed an appropriate statistical test for this and subsequent experiments, as it is useful in assessing the linear relationship between related pairs of interval data. While no formal NRMSE test was done, it nonetheless allows for quantitative assessment of T_2_ quantification quality and easy comparison with results from other approaches. NRMSE is reported ± 1 standard deviation (s.d.); Pearson’s r was deemed significant in accordance with corresponding *P* values, α = 0.001, 0.01, and 0.05. NRMSEs within tissues of interest of a given scan were also multiplied by mean T_2_ values within the tissue of interest of that patient, generating T_2_ value equivalents of error rates.

To more specifically evaluate the utility of the ROI-specific loss component, two loss function configurations from the ablation study were further analyzed at all R: no ROI-specific loss component ($\lambda_{{L_{1,\phi } }} = 0; { }\lambda_{{L_{1} }} ,{ }\lambda_{SSIM} ,{ }\lambda_{Feature} \ne 0$) and no ROI-specific or feature-based components ($\lambda_{{L_{1,\phi } }} ,\lambda_{Feature} = 0; { }\lambda_{{L_{1} }} ,{ }\lambda_{SSIM} \ne 0$). These models were intended to represent baselines in which all loss functions were preserved except the ROI-specific component, and a standard reconstruction loss function of pixel and SSIM-based loss components, respectively. Pearson’s r—evaluated in tissues of interest and globally—was calculated to determine the degree and significance of correlation between predicted maps and ground truth, both globally and within tissues of interest, α = 0.001, 0.01, and 0.05.

### Evaluation of accelerated acquisition scheme performance

Three versions of our pipeline (full pipeline, “No RNN,” and “Reduced Parameters”) were compared to state-of-the-art CS, DL, and DL/model-based solutions. At each R, MANTIS (54,413,056 trainable parameters) and MANTIS-GAN (54,413,056 [Generator] and 2,763,648 [Discriminator] trainable parameters) pipelines were trained using published network architectures, loss functions and undersampling strategies^42,43^. Loss function weightings for both were optimized through grid hyperparameter searches yielding the following: (MANTIS) $\lambda_{data}$ = 0.1, $\lambda_{cnn}$ = 1; (MANTIS-GAN) $\lambda_{data}$ = 0.1, $\lambda_{cnn}$ = 1, $\lambda_{GAN}$ = 0.01. To apply CS reconstruction, original MAPSS T_2_-prepared images were Fourier transformed into coil-combined k-space, 1D-inverse Fourier transformed along the readout direction, and individual slices in $k_{y} - k_{z}$ reconstructed using an $L_{1}$ wavelet-based algorithm with regularization coefficient 0.001^61^. CS reconstructed images were registered to the TE = 0 ms echo time image using a 3D rigid registration algorithm with a normalized mutual information criterion and fitted using Levenberg–Marquardt fitting to yield $T_{2}$ maps. Performance of these approaches and our proposed methods was evaluated through the following:

### Comparison of global and ROI-specific performance

To test for completeness of training, performance of our proposed pipelines was compared against state-of-the-art models that did not use ROI-specific components in predicting T_2_ maps. Pearson’s r (α = 0.001, 0.01, and 0.05) was used to compare model performances and assess strength of correlations to ground truth T_2_.

### Standard reconstruction metrics

Performance was reported in tissues of interest with standard reconstruction metrics: NRMSE (mean ± 1 s.d.) and Pearson’s r (α = 0.001, 0.01, and 0.05). NRMSEs were also converted into T_2_ value equivalents by tissue compartment as in the ablation study.

### T_2_ value retention

Fidelity of predicted maps to ground truth T_2_ was also assessed. First, predicted and ground truth T_2_ values were compared across tissues of interest within the test set (mean ± 1 s.d.), generating violin plots for all three anatomies with overlaid boxplots for T_2_ value distribution comparison. T_2_ agreement was also assessed through Bland–Altman analysis.

### Texture retention

Gray Level Co-Occurrence Matrix (GLCM)^62^ metrics were used to assess texture retention within tissues of interest. GLCM contrast and dissimilarity are maximized by large local pixel value changes and thus by sharper textures. GLCM homogeneity is maximized by small local pixel value changes, while GLCM energy and angular second moment (ASM) are maximized by few total pixel values within an image; hence, all three are maximized by smoothness. For each anatomy and R, we calculated these texture metrics at 4 orientations (θ = 0°, 45°, 90° and 135°; d = 1 pixel) and averaged across all orientations. Finally, we calculated intraclass correlation coefficients (ICCs) for all metrics with respect to ground truth (two-way mixed effects, single rater^63^) and reported 95% ICC confidence intervals (α = 0.001, 0.01, and 0.05). These tests were chosen as appropriate, as they assess both reliability and agreement of associated metrics, and in this use case, individual GLCM metric values themselves are considered the only rater, justifying the ICC test type selected.

### Repeatability study

To assess the robustness of pipelines to different datasets, two additional splits of the knee, hip and spine datasets were made, ensuring no patient was part of multiple validation and/or test datasets and that all scans from a given patient were only in one of training, validation and test for each split (folds 2 and 3 in Supplementary Table S2, where fold 1 is the original split). Additional hyperparameters searches optimized loss function weights on the two new splits. Optimized loss weights and corresponding T_2_ quantification and texture retention performance for each splits is presented at all tested R in the same manner as for the primary split.

### Raw multicoil data assessment

An in-house pipeline was developed that leveraged GE Orchestra 1.10 and other postprocessing tools to reconstruct coil-combined images from raw k-space data. As a proof of concept, knee MAPSS scans were performed on 3 volunteers, hip scans for 2, and lumbar spine for 2, all using the acquisition parameters listed for the retrospective datasets used for algorithm training, with raw k-space data saved for all. Multicoil k-space data (after ARC for knee and hip) was undersampled with the same center-weighted Poisson disc pattern described earlier, with each coil seeing the same undersampling pattern and k_y_-k_z_ lines being shared across different T_2_ weighted echo time k-spaces as previously described. Coil-combined images resulting from undersampled multi-coil data at all tested R were fed through corresponding post-processing pipelines to predict T_2_ map appearance. A radiologist with 2 years of experience segmented knee cartilage, hip cartilage, and intervertebral discs from these acquisitions, allowing for visualizations of predicted T_2_ maps and NRMSE calculations in ROIs.

## Results

### Ablation study results

Voxel-wise performance metrics for ablation study models at R = 8 are shown in Supplementary Table S3, with T_2_ value NRMSE equivalents in Supplementary Table S4. Within the knee and hip, all loss components were necessary to obtain the optimal combination of high Pearson’s r and low NRMSE in cartilage. For the lumbar spine, while all loss components proved vital in maximizing Pearson’s r and minimizing NRMSE in IVDs, performance improved when the initial recurrent network was omitted. Though quantitative analysis is shown for all three pipeline versions in subsequent experiments, the full model is designated as best for knee and hip, and the no RNN for the spine.

ROI-specific and global assessments of best models and corresponding models trained without an ROI-specific loss (λ_1,ϕ_ = 0) and models trained with a generic loss (λ_1,ϕ_ = 0, λ_Feat_ = 0) are shown in Supplementary Table S5. In the knee and hip, across nearly all R, ROI-specific loss addition leads to improved correlations between predicted and ground truth cartilage T_2_, with diminished performance globally. In the lumbar spine, which was trained with a substantially fewer batches than the knee and hip pipelines, these trends were inconsistent across tested R. Example predictions and ground truth for one slice of a patient in each pipeline are shown in Supplementary Fig. S3, showing that patterns of local T_2_ value elevations in cartilage and IVDs are better preserved with an ROI-specific loss as opposed to pipelines trained without the loss component.

### Visuals of network performance and comparison with state-of-the-art models

**Figure 2:**
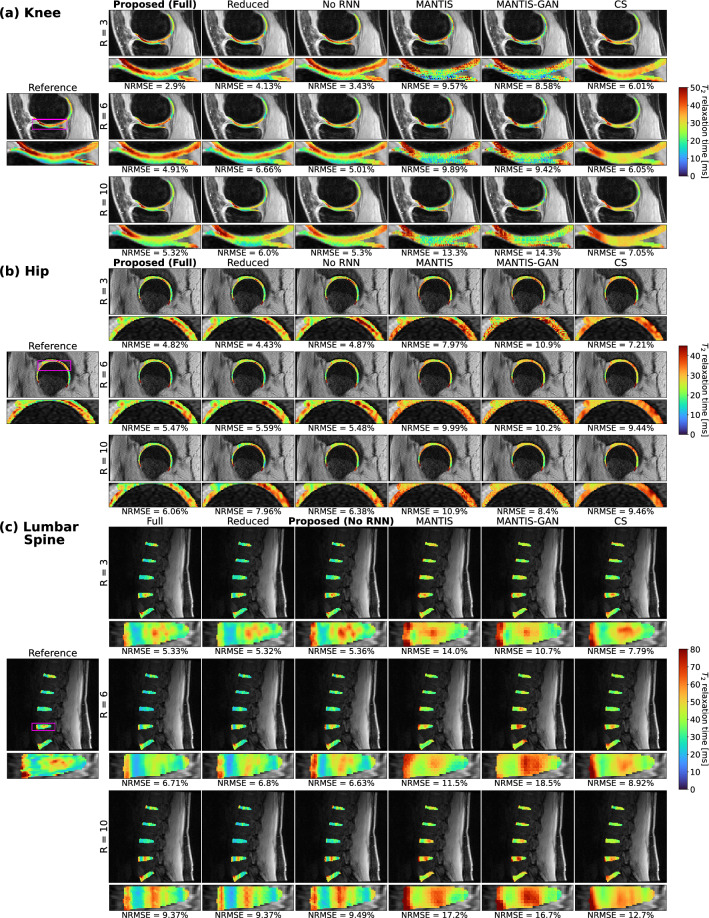
Comparison of predicted T_2_ maps with ROI-specific methodologies to past approaches. (**a**) Predicted T_2_ maps in knee cartilage for a representative patient within test set. T_2_ quantification performance was best in pipelines trained with ROI-specific losses (Full Model, Reduced Parameters, and No RNN), where strong fidelity to T_2_ values and patterns of local elevations within cartilage were maintained through R = 10, while other tested approaches did a poorer job in predicting T_2_ values in these maps. (**b**) Predicted hip cartilage T_2_ maps showed similar trends, where performance of the full model was especially strong, showing low T_2_ quantification error and better retaining local T_2_ elevations through R = 10 than other approaches. (**c**) Predicted T_2_ maps in lumbar spine IVDs show higher T_2_ quantification errors than in hip and knee cartilage, but ROI-specific loss pipelines best preserved map textures and values.

**Figure 3:**
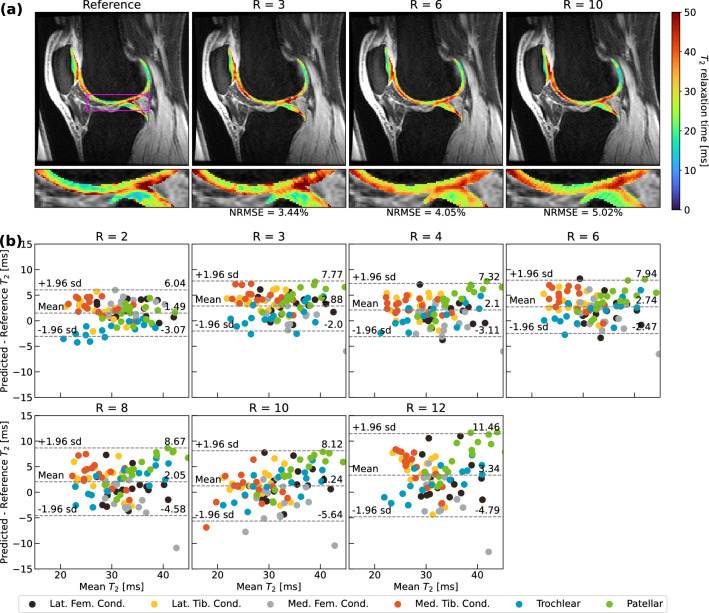
T_2_ quantification performance of optimal ROI-specific pipeline in knee cartilage. (**a**) Visual pipeline performance within the knee for a representative patient, with corresponding NRMSEs for cartilage in the predicted T_2_ map slice. Performance remains strong through R = 10, maintaining T_2_ patterns in the medial tibiofemoral cartilage, indicating pipeline utility. Predicted maps generated by the network are masked using a cartilage segmentation mask and superimposed on the ground truth, fully sampled TE = 0 ms MAPSS echo time image. (**b**) Bland–Altman plots for all scans within test set for which multiclass cartilage compartment segmentations were available (n = 16, 6 cartilage compartments for each). Predicted T_2_ values demonstrate minimal bias and tight limits of agreement across most tested R, with best performance coming from patellofemoral cartilage.

**Figure 4:**
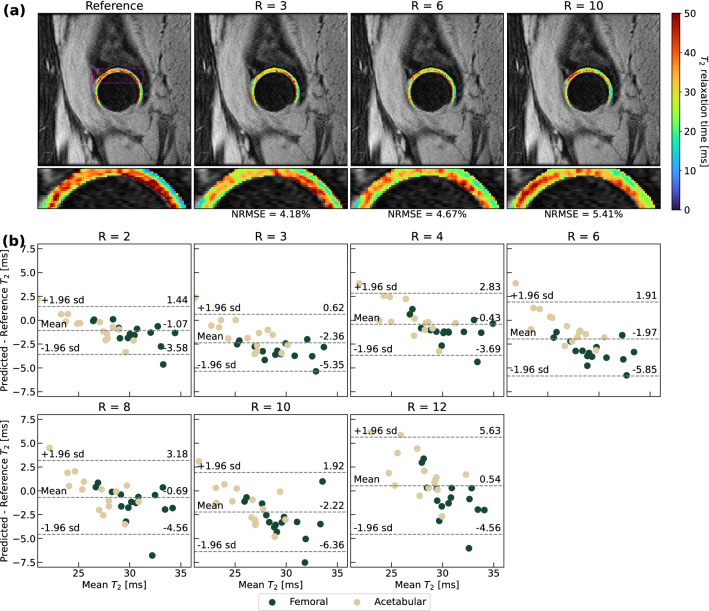
T_2_ quantification performance of optimal ROI-specific pipeline in hip cartilage. (**a**) Visual pipeline performance within the hip for a representative patient, with corresponding NRMSEs for cartilage in the predicted T_2_ map slice. Predicted maps are masked using a cartilage segmentation mask and superimposed on the ground truth, fully sampled TE = 0 ms MAPSS echo time image. For this patient, T_2_ patterns maintain through R = 10, although local T_2_ elevations are more diffusely predicted at higher R. (**b**) Bland–Altman plots for all scans within test set (n = 15, 2 cartilage compartments for each). Plots demonstrate very limited bias and even tighter limits of agreement from R = 2 through R = 12 than knee pipeline, showing hip pipeline effectiveness in reproducing T_2_ values from accelerated MAPSS acquisitions.

**Figure 5:**
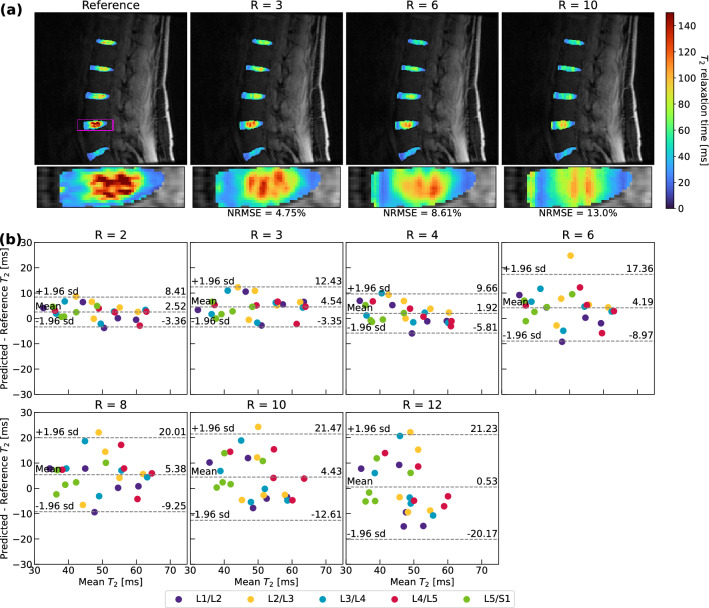
T_2_ quantification performance of optimal ROI-specific pipeline in lumbar spine intervertebral discs. (**a**) Visual pipeline performance within the lumbar spine IVDs for a representative patient, with corresponding NRMSEs for IVDs in the predicted T_2_ map slice. Predicted maps are masked using an IVD segmentation mask and superimposed on the ground truth, fully sampled TE = 0 ms MAPSS echo time image. Network performance is best through R = 6, after which local T_2_ elevations are diffuse and underestimated. (**b**) Bland–Altman plots for all scans within test set (n = 5, 5 IVDs plotted for each if segmentation of disc available). T_2_ value predictions reflect some bias and fairly wide limits of agreement, particularly above R = 4. These results indicate progress but the need for improvement. Smaller lumbar spine dataset and test set size are likely responsible for poorer model when compared to hip and knee performance, as well as the relatively smaller number of slices in k_z_, which exacerbates undersampling effects.

Predicted T_2_ maps are displayed at select R for knee, hip and lumbar spine models in Fig. 2 for our three pipelines and three methods from the literature. In knee, hip, and lumbar spine, T_2_ quantification performance is strongest with our proposed methods, maintaining low error rates, showing promising results compared with state-of-the-art methods through R = 10. Optimal architecture performances are further explored in Figs. 3–5**.** As shown in Fig. 3a, predicted T_2_ knee maps retained strong fidelity to ground truth within tibiofemoral joint cartilage. Patterns within predicted maps became slightly more diffuse as R increased to 10, as indicated by a slight rise in NRMSE for cartilage in the slice, but visually, T_2_ values and map patterns are preserved. As seen in Fig. 4a, hip predicted maps preserve T_2_ values well in femoral and acetabular cartilage through R = 10, although T_2_ patterns become more diffuse by R = 10. Figure 5a shows T_2_ map predictions in the lumbar spine. The L4-L5 IVD is shown in more detail, where T_2_ quantification performance was acceptable at R = 3, moderate at R = 6, and worse at R = 10, as indicated by rising IVD NRMSEs.

ROI and global performance comparisons of our selected pipelines against state-of-the-art approaches are in Supplementary Table S5. Across piplines trained with relatively large dataset (knee and hip), DL and model-based approaches (MANTIS and MANTIS-GAN) outperformed our proposed pipeline globally, but within cartilage ROIs, our pipeline exhibited stronger Pearson’s r at each tested R. These trends were not as strong in the lumbar spine pipelines, possibly owing to the randomness of training with a smaller dataset. Global and ROI-specific T_2_ predictions are further visualized in Supplementary Fig. S4, showing predicted T_2_ values exhibit substantially more visual fidelity to ground truth and lower NRMSE in state-of-the-art models compared to our pipeline, but a reversal of that trend in cartilage. In the lumbar spine, at some but not all R, those trends held, yielding similar conclusions to the Pearson’s r analysis.

### Evaluation of T_2_ quantification performance and comparison with state-of-the-art models

#### Voxel-wise T_2_ evaluation fidelity

Pearson’s r and NRMSE across all anatomies and R for our approaches and state-of-the-art methods are in Table 2. T_2_ value NRMSE equivalents are in Supplementary Table S6. For all anatomies and across nearly all R, T_2_ quantification performance is strongest in our methods, particularly in the No RNN and full model pipelines, compared to state-of-the-art models.

**Table 2:** ROI-specific model performance in standard metrics from R = 2 through R = 12.

Tissue	R	Metric	Full model	Reduced parameters	No RNN	MANTIS	MANTIS-GAN	CS
Knee cartilage	2	NRMSE	5.52 ± 1.25	6.07 ± 3.21	**4.76 ± 1.78**	14.4 ± 2.85	13.5 ± 3.3	8.92 ± 3.2
		Pearson's r	0.748***	0.736***	0.807***	0.587***	0.611***	0.620***
	3	NRMSE	6.52 ± 2.17	7.18 ± 3.08	**6.39 ± 2.59**	16.5 ± 3.43	15.1 ± 2.89	9.92 ± 3.23
		Pearson's r	0.695***	0.668***	0.722***	0.467***	0.502***	0.559***
	4	NRMSE	**7.54 ± 2.96**	9.56 ± 5.47	7.56 ± 3.19	16.6 ± 3.73	15.7 ± 4.5	11.8 ± 3.73
		Pearson's r	0.651***	0.637***	0.677***	0.451***	0.467***	0.486***
	6	NRMSE	**8.09 ± 2.65**	10.7 ± 6.67	8.44 ± 3.49	15.2 ± 2.33	16.3 ± 3.91	12.4 ± 4.1
		Pearson's r	0.612***	0.610***	0.629***	0.397***	0.378*	0.445***
	8	NRMSE	**8.94 ± 2.66**	9.59 ± 3.83	10.1 ± 4.42	16.7 ± 2.73	17.3 ± 2.39	12.9 ± 3.93
		Pearson's r	0.585***	0.574***	0.609***	0.352***	0.364**	0.410***
	10	NRMSE	9.77 ± 3.44	10.2 ± 3.61	**9.35 ± 3.5**	17.6 ± 2.48	16.7 ± 3.44	13.4 ± 3.76
		Pearson's r	0.555***	0.514***	0.565***	0.327***	0.333***	0.386***
	12	NRMSE	10.7 ± 2.32	**9.93 ± 3.76**	10.5 ± 3.37	18.2 ± 4.5	20.5 ± 5.58	13.4 ± 3.96
		Pearson's r	0.491***	0.545***	0.511***	0.290***	0.287***	0.381***
Hip cartilage	2	NRMSE	3.97 ± 1.03	4.1 ± 1.1	**3.79 ± 0.807**	4.58 ± 0.993	8.21 ± 1.42	14.8 ± 2.78
		Pearson's r	0.794***	0.782***	0.770***	0.716***	0.514***	0.310***
	3	NRMSE	6.53 ± 1.63	5.63 ± 1.68	**5.25 ± 1.13**	6.41 ± 1.31	10.0 ± 1.57	12.9 ± 3.15
		Pearson's r	0.705***	0.726***	0.703***	0.596***	0.372***	0.332***
	4	NRMSE	6.15 ± 1.01	6.17 ± 1.47	**5.84 ± 0.891**	7.33 ± 1.67	9.97 ± 1.74	11.8 ± 2.03
		Pearson's r	0.646***	0.665***	0.648***	0.510***	0.333***	0.339***
	6	NRMSE	8.1 ± 1.85	8.22 ± 2.06	**7.48 ± 1.52**	8.63 ± 2.32	9.68 ± 1.92	11.8 ± 2.14
		Pearson's r	0.587***	0.597***	0.570***	0.382***	0.321***	0.334***
	8	NRMSE	6.97 ± 1.93	**6.33 ± 1.33**	6.98 ± 1.45	10.2 ± 2.72	12.0 ± 2.64	10.5 ± 2.3
		Pearson's r	0.598***	0.588***	0.558***	0.334***	0.237***	0.347***
	10	NRMSE	8.99 ± 2.65	**8.12 ± 1.28**	8.7 ± 3.46	9.74 ± 2.24	10.5 ± 1.91	10.2 ± 2.4
		Pearson's r	0.558***	0.534***	0.522***	0.279***	0.268***	0.335***
	12	NRMSE	7.75 ± 1.5	8.27 ± 2.19	**7.34 ± 1.38**	9.74 ± 2.23	11.5 ± 2.36	10.3 ± 2.52
		Pearson's r	0.517***	0.566***	0.512***	0.280***	0.228***	0.349***
Lumbar spine IVD	2	NRMSE	6.71 ± 1.7	6.86 ± 1.57	**4.86 ± 1.16**	8.78 ± 2.08	8.95 ± 1.91	10.1 ± 3.06
		Pearson's r	0.865***	0.866***	0.884***	0.784***	0.785***	0.802***
	3	NRMSE	9.92 ± 2.39	8.76 ± 2.16	**7.13 ± 1.69**	11.0 ± 1.17	11.3 ± 1.74	9.48 ± 1.4
		Pearson's r	0.836***	0.823***	0.832***	0.717***	0.712***	0.777***
	4	NRMSE	10.3 ± 3.02	9.73 ± 3.07	**7.42 ± 1.1**	12.1 ± 1.24	12.6 ± 1.35	11.3 ± 2.31
		Pearson's r	0.799***	0.813***	0.819***	0.680***	0.671***	0.723***
	6	NRMSE	12.1 ± 3.58	12.2 ± 4.11	**10.3 ± 3.31**	15.6 ± 2.65	12.1 ± 1.9	12.0 ± 2.76
		Pearson's r	0.776***	0.764***	0.771***	0.660***	0.658***	0.728***
	8	NRMSE	13.4 ± 3.89	13.0 ± 2.63	**12.0 ± 3.07**	13.2 ± 1.42	12.7 ± 1.7	12.8 ± 2.53
		Pearson's r	0.742***	0.723***	0.742***	0.631***	0.645***	0.695***
	10	NRMSE	15.3 ± 3.22	14.8 ± 2.78	14.8 ± 2.26	13.8 ± 1.57	**13.2 ± 1.81**	15.0 ± 3.77
		Pearson's r	0.695***	0.700***	0.672***	0.647***	0.636***	0.648***
	12	NRMSE	18.1 ± 1.95	23.1 ± 2.71	18.8 ± 2.76	14.8 ± 3.03	**14.1 ± 1.88**	24.8 ± 11.2
		Pearson's r	0.664***	0.320***	0.643***	0.651***	0.614***	0.586***

Top performing pipeline for each metric, at each R, is shown in bold.

Performances of pipelines trained with ROI-specific losses and other state-of-the-art methods in T_2_ quantification error rates in knee cartilage, hip cartilage, and lumbar spine IVDs. NRMSEs are reported ± 1 s.d., and Pearson’s r is reported with significances as follows: *P < 0.05, **P < 0.01, ***P < 0.001 (knee: n = 90; hip: n = 15; lumbar spine: n = 5). Across all anatomies, performances were strongest in ROI-specific loss pipelines (Full Model, Reduced Parameters, and No RNN): in the knee, the No RNN and Full Model pipelines particularly excelled across all tested R; in the hip, the No RNN pipeline was strong in maintaining minimal T_2_ quantification errors, while the Full Model and Reduced Parameters models had strongest correlations between predicted maps and ground truth; in the lumbar spine, the No RNN pipeline especially had strong T_2_ quantification performance. Performance in the knee and hip pipelines is strong and below clinically significant T_2_ changes across nearly all tested R, while Pearson’s r indicates strong T_2_ value preservation in the lumbar spine through R = 6. T_2_ quantification performance is thus promising in all three pipelines, but particularly for the knee and hip.

An exhaustive examination of knee T_2_ quantification performance, stratified by cartilage compartments, is in Supplementary Tables S7 and S8. For the full model, across all cartilage compartments, T_2_ estimation errors remained under 10% through R = 10 across all cartilage compartments while Pearson’s r ranged from 0.748 at R = 2 to 0.491 at R = 12, indicating strong correlations^64^ between predictions and ground truth at R = 2 and moderate correlations through R = 12. For some cartilage compartments and R, performance was stronger in the No RNN pipeline. Interestingly, quantification performance was strongest in patellofemoral joint cartilage, generally exhibiting lower NRMSE and stronger correlations. Our ROI-specific loss pipelines outperformed state-of-the-art models in each cartilage compartment.

Supplementary Tables S9 and S10 show hip T_2_ quantification performance across cartilage compartments. As in the knee, quantification performance was strong, with error rates across all cartilage under 9% through R = 12 for the no RNN and full model pipelines. While the no RNN pipeline had stronger quantification errors, the full model had higher Pearson’s r, which ranged from 0.794 at R = 2 to 0.517 at R = 12, showing strong correlations between predictions and ground truth through R = 3 and moderate correlations through R = 12. T_2_ quantification performance was slightly stronger in femoral than acetabular cartilage. Our pipelines again outperformed state-of-the-art models in each cartilage compartment.

Supplementary Tables S11 and S12 show lumbar spine T_2_ quantification performance, which was mixed. Pearson’s r across all discs was very high, ranging from 0.884 at R = 2 to 0.643 at R = 12 for the no RNN model, indicating strong correlations through R = 8 and moderate correlations through R = 12 to ground truth. That said, IVD error rates were markedly higher across all R than in hip and knee cartilage, ranging from 4.86% to 18.8%. Though there was some volatility, error rates and Pearson’s r generally showed poorest T_2_ quantification in L1/L2 and L2/L3 discs. Through R = 8, ROI-specific loss pipelines outperformed state-of-the-art models at nearly all disc levels, with stronger Pearson’s r in most IVD levels through R = 12.

#### T_2_ Value retention on region of interest averages

Bland–Altman plots are provided for the knee, hip and lumbar spine in Figs. 3b, 4b, and 5b. In knee and hip, T_2_ values are predicted with minimal bias with respect to ground truth. The ± 1.96 s.d. limits of agreement were less than approximately ± 6 ms with mean biases under ± 3 ms through R = 8 for knee cartilage (Fig. 3b). Among cartilage compartments, predictions in trochlear and patellar cartilage showed the least bias, while tibiofemoral cartilage T_2_ was generally slightly overestimated. In the hip (Fig. 4b), ± 1.96 s.d. limits of agreement were less than approximately ± 5 ms with mean biases under ± 3 ms through R = 12, although T_2_ quantification performance was similar across femoral and acetabular cartilage. In the lumbar spine (Fig. 5b), limits of agreement were considerably wider than the hip and knee pipelines, particularly above R = 4. While the line of equality was contained in these limits at all R, spine pipelines generally overestimated T_2_ values. While at some particular R, a disc level saw poorer T_2_ quantification than others (i.e. L2/L3 at R = 6), on balance, predicted maps yielded similar bias and error across all discs.

Supplementary Fig. S5 shows T_2_ value distributions in violin and boxplots. Plots reveal minimal bias in hip cartilage predicted T_2_ maps and slight but limited bias towards overestimating T_2_ in knee cartilage. In the lumbar spine, more volatility was observed in predicted T_2_ distributions, likely due to small test set size (n = 5), but at least through R = 6, these deviations had limited magnitude.

#### Texture retention

ICCs ± 1 s.d. for GLCM metrics are in Table 3 for our best performing pipelines: no RNN and full model. In knee cartilage, ICCs showed significant correlations between predicted and ground truth GLCM metrics at all R for smooth textures and many R for sharp textures, indicating good to excellent reliability in preserving smooth textures (ASM and energy) at all R and moderate reliability in preserving sharper textures at low R (dissimilarity). In hip cartilage, ICCs showed significant correlations across all R in preserving smooth textures, and at low to moderate R for sharper textures. Reliability in smooth texture preservation ranged from good to excellent for all R and moderate for sharper textures at low to medium R. In both knee and hip cartilage, the full pipeline saw substantially higher GLCM ICCs for smooth and sharper texture across nearly all R. Within the lumbar spine, ICCs were significant across nearly all R for smoother textures. While ICCs were reasonable high for some R in contrast metrics, confidence intervals were wide, limiting findings of significant correlations. ICCs showed moderate to excellent reliability in preserving smoother textures, and poor to moderate reliability for sharper textures. For the spine, the No RNN model yielded optimal texture retention.

**Table 3:** Texture retention analysis in No RNN and Full Model pipelines.

		R	GLCM texture metric				
			Contrast	Dissimilarity	Homogeneity	ASM	Energy
Knee	Full model	2	0.307 ± 0.18**	0.638 ± 0.12***	0.734 ± 0.09***	0.966 ± 0.015***	0.954 ± 0.02***
		3	0.153 ± 0.2	0.521 ± 0.15***	0.735 ± 0.09***	0.962 ± 0.015***	0.95 ± 0.02***
		4	0.11 ± 0.2	0.387 ± 0.17***	0.61 ± 0.12***	0.973 ± 0.01***	0.95 ± 0.02***
		6	0.0667 ± 0.2	0.22 ± 0.19*	0.382 ± 0.17***	0.97 ± 0.015***	0.94 ± 0.025***
		8	0.061 ± 0.2	0.111 ± 0.2	0.0615 ± 0.2	0.952 ± 0.02***	0.9 ± 0.04***
		10	0.0594 ± 0.2	0.218 ± 0.19*	0.307 ± 0.18**	0.961 ± 0.015***	0.928 ± 0.03***
		12	0.0032 ± 0.2	− 0.066 ± 0.2	− 0.178 ± 0.19	0.927 ± 0.03***	0.861 ± 0.055***
	No RNN	2	0.455 ± 0.16***	0.599 ± 0.13***	0.32 ± 0.18***	0.898 ± 0.04***	0.904 ± 0.04***
		3	0.394 ± 0.17***	0.523 ± 0.15***	0.383 ± 0.17***	0.709 ± 0.11***	0.802 ± 0.07***
		4	0.262 ± 0.18**	0.305 ± 0.18**	0.244 ± 0.18**	0.646 ± 0.12***	0.754 ± 0.09***
		6	0.103 ± 0.2	0.0574 ± 0.2	0.061 ± 0.2	0.874 ± 0.045***	0.869 ± 0.05***
		8	0.0645 ± 0.2	0.0411 ± 0.2	0.0435 ± 0.2	0.922 ± 0.03***	0.911 ± 0.035***
		10	0.0474 ± 0.2	0.0382 ± 0.2	0.093 ± 0.2	0.92 ± 0.035***	0.913 ± 0.035***
		12	0.0568 ± 0.2	0.0315 ± 0.2	0.0885 ± 0.2	0.818 ± 0.065***	0.862 ± 0.055***
Hip	Full model	2	0.312 ± 0.34*	0.633 ± 0.23***	0.837 ± 0.12***	0.945 ± 0.04***	0.957 ± 0.035***
		3	0.369 ± 0.32*	0.671 ± 0.21***	0.816 ± 0.14***	0.976 ± 0.02***	0.98 ± 0.015***
		4	0.328 ± 0.33*	0.597 ± 0.25***	0.801 ± 0.15***	0.957 ± 0.035***	0.954 ± 0.04***
		6	0.235 ± 0.35	0.475 ± 0.3**	0.645 ± 0.23***	0.939 ± 0.05***	0.941 ± 0.045***
		8	0.199 ± 0.36	0.487 ± 0.28**	0.823 ± 0.13***	0.923 ± 0.06***	0.933 ± 0.055***
		10	0.127 ± 0.36	0.308 ± 0.34	0.48 ± 0.29**	0.862 ± 0.11***	0.855 ± 0.11***
		12	0.198 ± 0.36	0.38 ± 0.32*	0.523 ± 0.28**	0.927 ± 0.06***	0.914 ± 0.07***
	No RNN	2	0.285 ± 0.34	0.399 ± 0.32*	0.406 ± 0.31*	0.855 ± 0.11***	0.841 ± 0.12***
		3	0.15 ± 0.36	0.241 ± 0.35	0.292 ± 0.34	0.867 ± 0.1***	0.85 ± 0.12***
		4	0.113 ± 0.36	0.202 ± 0.36	0.282 ± 0.34	0.836 ± 0.12***	0.813 ± 0.14***
		6	0.0394 ± 0.36	0.0504 ± 0.36	0.0785 ± 0.36	0.793 ± 0.15***	0.767 ± 0.16***
		8	0.0229 ± 0.37	0.000593 ± 0.37	− 0.0583 ± 0.37	0.682 ± 0.21***	0.653 ± 0.22***
		10	− 0.00292 ± 0.36	− 0.0328 ± 0.37	− 0.196 ± 0.36	0.644 ± 0.23***	0.621 ± 0.24***
		12	− 0.00208 ± 0.37	− 0.0312 ± 0.36	− 0.0646 ± 0.36	0.712 ± 0.2***	0.687 ± 0.2***
Lumbar spine	Full model	2	0.557 ± 0.7	0.695 ± 0.62	0.744 ± 0.57*	0.892 ± 0.35**	0.923 ± 0.27**
		3	0.499 ± 0.73	0.615 ± 0.67	0.644 ± 0.66	0.819 ± 0.48*	0.872 ± 0.39*
		4	0.236 ± 0.8	0.421 ± 0.76	0.497 ± 0.73	0.67 ± 0.64	0.775 ± 0.54*
		6	0.341 ± 0.78	0.428 ± 0.76	0.262 ± 0.8	0.566 ± 0.7	0.67 ± 0.64*
		8	0.0633 ± 0.81	0.152 ± 0.8	0.276 ± 0.79	0.685 ± 0.62	0.728 ± 0.58*
		10	− 0.0393 ± 0.81	− 0.0631 ± 0.81	− 0.0699 ± 0.81	0.403 ± 0.76	0.479 ± 0.74*
		12	− 0.0697 ± 0.81	− 0.156 ± 0.8	− 0.424 ± 0.76	0.16 ± 0.8	0.198 ± 0.8*
	No RNN	2	0.496 ± 0.73	0.731 ± 0.58*	0.883 ± 0.37**	0.967 ± 0.14***	0.975 ± 0.11***
		3	0.357 ± 0.78	0.615 ± 0.67	0.807 ± 0.5*	0.909 ± 0.31**	0.934 ± 0.24**
		4	0.336 ± 0.78	0.607 ± 0.68	0.771 ± 0.54*	0.874 ± 0.38*	0.91 ± 0.31**
		6	0.307 ± 0.78	0.53 ± 0.72	0.604 ± 0.68	0.903 ± 0.32**	0.916 ± 0.29**
		8	0.2 ± 0.8	0.4 ± 0.76	0.59 ± 0.68	0.847 ± 0.44*	0.871 ± 0.39*
		10	0.0696 ± 0.81	0.184 ± 0.8	0.386 ± 0.76	0.692 ± 0.62	0.726 ± 0.59*
		12	0.0157 ± 0.82	0.0858 ± 0.81	0.325 ± 0.78	0.561 ± 0.7	0.591 ± 0.68*

Intraclass correlation coefficients (ICCs) of Gray Level Co-Occurrence Matrix (GLCM)-based metrics. Contrast and dissimilarity are most sensitive to sharper image textures, while homogeneity, ASM, and energy are most sensitive to smoother image textures. Significance in correlations is noted as follows: *P < 0.05, **P < 0.01, ***P < 0.001 (knee: n = 16; hip: n = 15; lumbar spine: n = 5). In the knee and hip, Full Model pipelines outperformed No RNN versions in retention of smooth and sharp textures. In the lumbar spine, the No RNN pipeline outperformed the Full Model version, possibly because the smaller lumbar spine dataset size made training a larger network with a multi-component loss more difficult. In conjunction with standard reconstruction metrics, the Full Model pipeline was selected as the best knee and hip model, whereas the No RNN pipeline was selected as the best lumbar spine model. Top models in all anatomies preserved smoother textures at nearly all tested R, while dissimilarity texture metrics showed sharper textures were significantly correlated with ground truth and preserved in the knee and hip at low to medium R. In the lumbar spine, mean ICCs for sharper textures at many tested R also were high, but small dataset size likely led to wide standard deviations that prevented significant conclusions from being reached. All told, many textures are preserved in T_2_ maps by all pipelines, particularly in the knee and hip.

### Repeatability study

Optimal loss weightings from hyperparameter searches on the two additional splits are in Supplementary Table S13. Results of trainings on additional splits in T_2_ quantification error, Pearson’s r, and texture metrics are in Supplementary Tables S14, S15, S16. In the knee and hip pipelines, experiments show comparable results across all folds for these metrics. In the lumbar spine, Pearson’s r exhibited similar values across all folds, but in some cases, mean texture metric ICCs and NRMSEs exhibited substantial differences. However, confidence intervals were very wide for ICCs and NRMSEs in the lumbar spine, likely due to limited test set size (n = 5).

### Raw multicoil data assessment

Supplementary Fig. S6 shows T_2_ maps predicted from our proposed pipelines on retrospectively undersampled raw k-space data. In the knee, T_2_ quantification errors were low through R = 12, with local T_2_ elevations preserved and little dip in performance compared to corresponding retrospectively undersampled coil-combined knee data. In the hip, T_2_ quantification errors were low, with local T_2_ elevations reproduced at most R; while performance at higher R matched expected performance from coil-combined experiments, lower R quantification errors were slightly higher. Performance was more volatile in the lumbar spine, where through R = 4, T_2_ quantification errors matched expected results and local T_2_ patterns were generally preserved, but performance degraded substantially above R = 4.

### Discussion and conclusions

In this work, we present data-driven pipelines that leverage recurrent UNet architectures and multi-component losses to accelerate MAPSS T_2_ mapping for anatomies where a subset of tissues is of particular clinical interest. By image processing and standard reconstruction metrics, through R = 10, our knee pipelines retained fidelity to T_2_ values with tight limits of agreement, preserving smooth textures with good to excellent reliability and sharper ones with moderate reliability for most tested R. While the no RNN pipeline delivered lower NRMSEs and higher Pearson’s r across many cartilage compartments and R than full model, its texture retention was poorer, making the full model better suited to preserve small, key diagnostic features. In hip cartilage, predicted maps retained T_2_ fidelity through R = 12 with tight limits of agreement, preserved smooth textures with good to excellent agreement across tested R, and maintained sharper textures at low to moderate R. As with the knee, texture retention was strongest in the full pipeline despite lower no RNN NRMSEs. In IVDs, the no RNN pipeline delivered best standard reconstruction metric and texture retention performance. Despite maintaining smoother textures with moderate to excellent agreement across tested R and preserving sharper textures at lower R, the IVD pipeline revealed biases and fairly wide limits of agreement in T_2_ preservation, particularly at R = 6 and higher. When assessed on retrospectively undersampled multicoil raw k-space data, the knee and hip pipelines saw minimal degradation in performance as compared to results from images undersampled via synthetic k-space, whereas the lumbar spine pipeline exhibited similar performance through R = 4. Furthermore, repeatability studies indicated that, particularly for the hip and knee, performance was stable with respect to datasets. All told, these metrics indicate promise for the knee and hip pipelines in MAPSS T_2_ mapping acceleration, and progress but room for improvement in IVDs.

Assessments of ROI-specific loss component utility showed its potential for improving predictions in accelerated acquisition schemes. When trained with sufficiently large datasets, as our knee and hip pipelines were, its inclusion saw stronger fidelity to local T_2_ patterns in cartilage ROIs and reduced T_2_ quantification errors compared to analogous pipelines trained without the ROI-specific loss component. Compared to state-of-the-art DL pipelines, knee and hip pipelines saw improved Pearson’s r in cartilage ROIs but poorer global Pearson’s r, as expected from the focused training approach. Interestingly, CS approaches exhibit relatively strong NRMSEs while generating relatively smooth predicted T_2_ maps; this is possibly because in training, DL-based approaches simultaneously removed aliasing artifacts and performed T_2_ fitting, and could attempt to preserve finer details than a CS approach performing those steps sequentially. While our approaches outperformed state-of-the-art methods at many R and tissue compartments in the lumbar spine, global Pearson’s r indicated this may have been partially due to some models being more completely trained than others. These results may have been different with a larger lumbar spine training set. Nonetheless, the value of ROI-specific loss functions in accelerated acquisition pipelines is clear: with sufficiently large datasets, they can optimize for ROIs and outperform state-of-the-art approaches at high R, as existing approaches are optimized for global and not ROI-specific performance.

We can contextualize performance by comparing quantification errors to clinically significant T_2_ changes. In the knee, T_2_ increases 13.4% in lateral femoral condyle (LFC) cartilage, 12.3% in medical femoral condyle (MFC) cartilage, and 8.1% in medial tibial condyle (MTC) cartilage among patients with mild OA compared to controls^65^. Our top-performing knee pipeline saw errors below this benchmark through R = 12 in the LFC and at R = 2 in the MTC. In IVDs, T_2_ decreases 36.3% in the nucleus pulposus and 24.2% in the annulus fibrosus from healthy to degenerative discs^66^. Our top-performing pipeline saw quantification errors for each disc below the more stringent 24.2% through R = 12. In the hip, T_2_ values among healthy patients that progress to OA within 18 months are 7.3% higher in femoral and 5.2% higher in acetabular cartilage compared to controls^67^. Our top-performing hip pipeline had errors below these benchmarks at all R in femoral cartilage and at R = 2 in acetabular cartilage. Clinical metrics thus depict promise for pipelines in all three anatomies in maintaining sub-clinical-significance quantification errors.

Clinical and standard metrics show knee and hip pipeline performances to be particularly promising—the T_2_ values, map texture preservation, and error rates relative to clinical benchmarks all mark meaningful progress towards reducing cMRI acquisition time for eventual clinical use. That said, while lumbar spine performance was strong by clinical metrics, it lagged the knee and hip by standard reconstruction metrics. One explanation is dataset size: the lumbar spine dataset had substantially fewer scans and imaging slices than the knee and hip. This has twofold impact: (1) the strength of a model trained from a smaller dataset is inherently limited, and (2) having only 5 test set scans limits statistical power and induces wide standard deviations of metrics, preventing significant conclusions from being reached. The effects of this small dataset size particularly surface in repeatability studies. Furthermore, lumbar spine acquisitions were more susceptible to breathing artifacts and had fewer slices than the hip and knee; undersampling therefore left fewer lumbar spine k_y_-k_z_ lines sampled compared to the hip and knee, inducing worse initializations and possibly poorer performance. Nonetheless, to our knowledge, this is the first DL application to accelerate lumbar spine cMRI, marking progress that must be furthered with additional data procurement and algorithm development for clinical utility.

The GLCM-based textural retention evaluation demonstrated a framework through which reconstruction performance can be better evaluated than through standard metrics like SSIM, NRMSE, and PSNR. ICCs of GLCM metrics between predicted and ground truth T_2_ maps allow for intuitive, scaled measurements that can reflect how well a particular texture was preserved: for example, visual inspection of predicted T_2_ maps in knee and hip cartilage in Figs. 3, 4 indicate that sharp textures are preserved better by the hip pipeline. This qualitative observation is confirmed by the GLCM Dissimilarity ICCs observed for the full model in the hip and knee pipelines in Table 3 at several tested R. This work could be furthered by extending this analysis to additional GLCM metrics for an even more thorough assessment of textural feature retention. Additional future improvements could also include pre-processing cartilage and IVD tissues prior to GLCM metric calculation to improve stability of these metrics, as other groups have started to do^68^.

Moreover, by showing results at 7 acceleration factors instead of the 2–3 typical in the literature, we found performance did not always degrade steadily as R increased. Networks therefore may be sensitive not just to general undersampling patterns, but also the specific nature of the pattern. Thus, when future DL reconstruction pipelines are trained, a library of undersampling patterns may be advisable to encourage robustness to sampling patterns^69^.

This study has limitations. First, we used retrospectively undersampled coil-combined magnitude echo time images that, in the knee and hip, had undergone ARC processing in their reconstruction, with 4 edge slices discarded for all data. Due to coil combination and post-processing, the k-space being undersampled would not match the acquisition’s multi-coil k-space. Additionally, while we undersampled the MAPSS acquisition ellipse for each anatomy, the hip acquisitions had ‘no phase wrap’ applied, meaning that tested undersampling patterns would differ from those implemented on the scanner. While our raw k-space experiments show performance degradation was limited compared to coil-combined magnitude image experiments, models would be stronger if trained with a similarly sized multicoil k-space dataset. Second, this network is specific to our sampling patterns and acquisition parameters, and new pipelines would need to be trained should parameters like MAPSS T_2_ echo times be substantially changed. Finally, the lumbar spine dataset size is rather small, limiting the power of conclusions.

To conclude, this study shows a novel means of training DL pipelines to accelerate cMRI in anatomies where specific tissues are of heightened clinical importance. In knee and hip, pipelines were effective at high R in maintaining textures, keeping fidelity to T_2_ values, and minimizing T_2_ quantification errors, whereas in the lumbar spine, the pipeline performed reasonably by those same criteria, but poorer in T_2_ value fidelity and quantification errors. This reflects progress towards clinically useful pipelines that specialize in MSK T_2_ mapping. The GLCM-based textural retention analysis elucidates an alternate to standard reconstruction metrics, allowing for intuitive measures of the types of features best preserved by a accelerated acquisition schemes, potentially allowing for better quantitative assessment of model performance. Future directions include multicoil k-space training, simultaneous MAPSS T_1ρ_ and T_2_ acceleration, and temporal undersampling of T_2_ weighted echo time images.

## Supplementary Information

Supplementary Information.

## Data Availability

The datasets analyzed during the current study have been collected as part of multi-year studies or volunteers scans at the UCSF and their public release is not currently possible due to data privacy concerns. Codes to reproduce the results of this work are available upon reasonable request from the corresponding author (A. Tolpadi).
