# Supplementary material for: Region of interest-specific loss functions improve T2 quantification with ultrafast T2 mapping MRI sequences in knee, hip and lumbar spine
Source: Sci Rep. 2022 Dec 23;12:22208. doi: 10.1038/s41598-022-26266-z (PMC9789075; doi:10.1038/s41598-022-26266-z)
Supplement: Supplementary file 1 — Supplementary Information. [file 41598_2022_26266_MOESM1_ESM.pdf]

Region of Interest-Specific Loss Functions Improve Predicted T_2 Map Quality in Ultrafast T_2 Mapping in Knee, Hip and Lumbar Spine

Aniket A. Tolpadi[1*], Misung Han[1], Francesco Calivà[1], Valentina Pedoia[1], Sharmila Majumdar[1]
[1]Department of Radiology and Biomedical Imaging, University of California, 1700 4th Street, San Francisco, CA 94158, United States of America
*Correspondence should be addressed to A.T. (email: Aniket.Tolpadi@ucsf.edu)

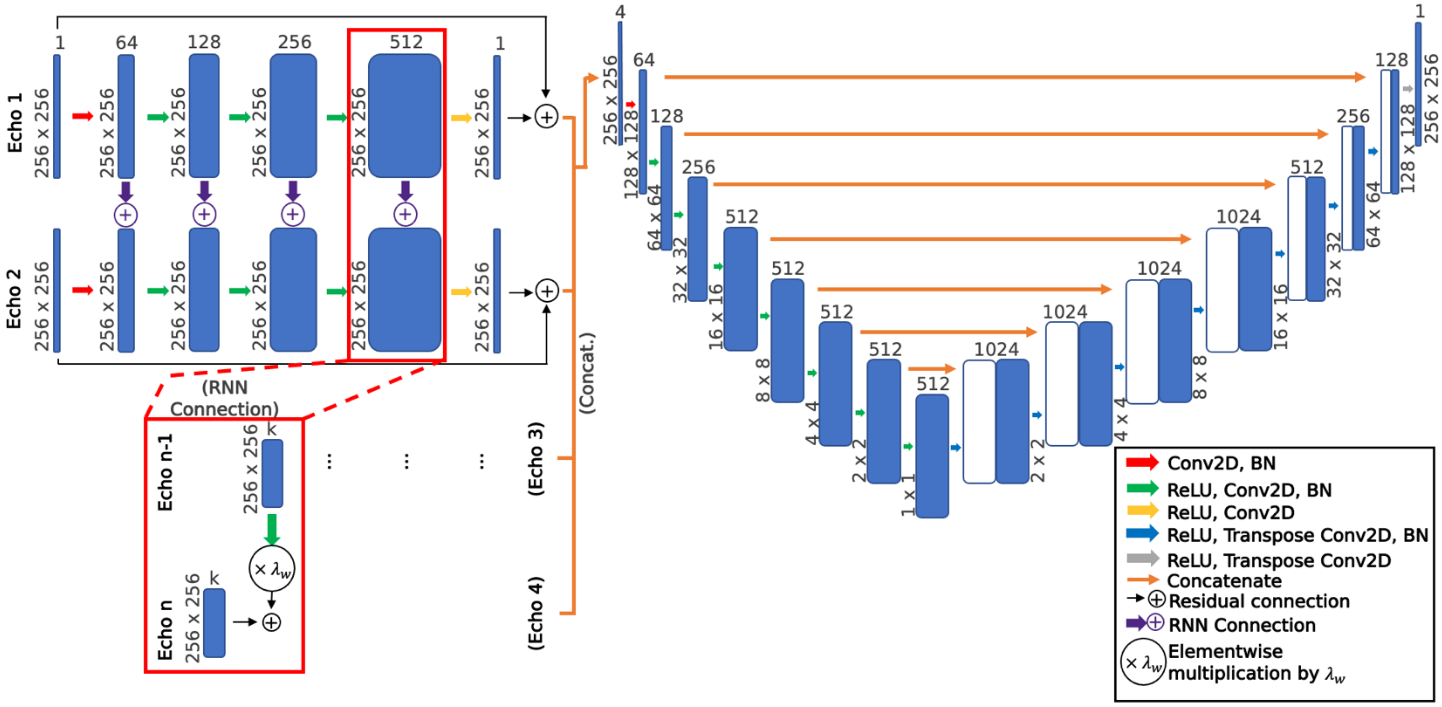

Supplementary Information Figure S1: Network architecture. Recurrent UNet network to predict T_2 map appearance from spatially undersampled T_2-specific MAPSS acquisition echo time images. T_2 weighted images at each echo time have a unique, 5-layer processing stream, with information passed between corresponding layers in adjacent temporal processing streams through RNN connections: ReLU, 2D convolutional layer, batch normalization, and elementwise multiplication by weighting parameter λ_w=0.2 before being added to corresponding layers of the next stream. Processing stream outputs are concatenated and fed to the UNet, which predicts T_2 maps. Depths and dimensions are provided at each layer. This schematic reflects the "full model"; additional versions were trained without the initial RNN and solely with the UNet network (No RNN) and a streamlined version in which the depth of each layer was half what is depicted in this full model schematic (Reduced Parameters).

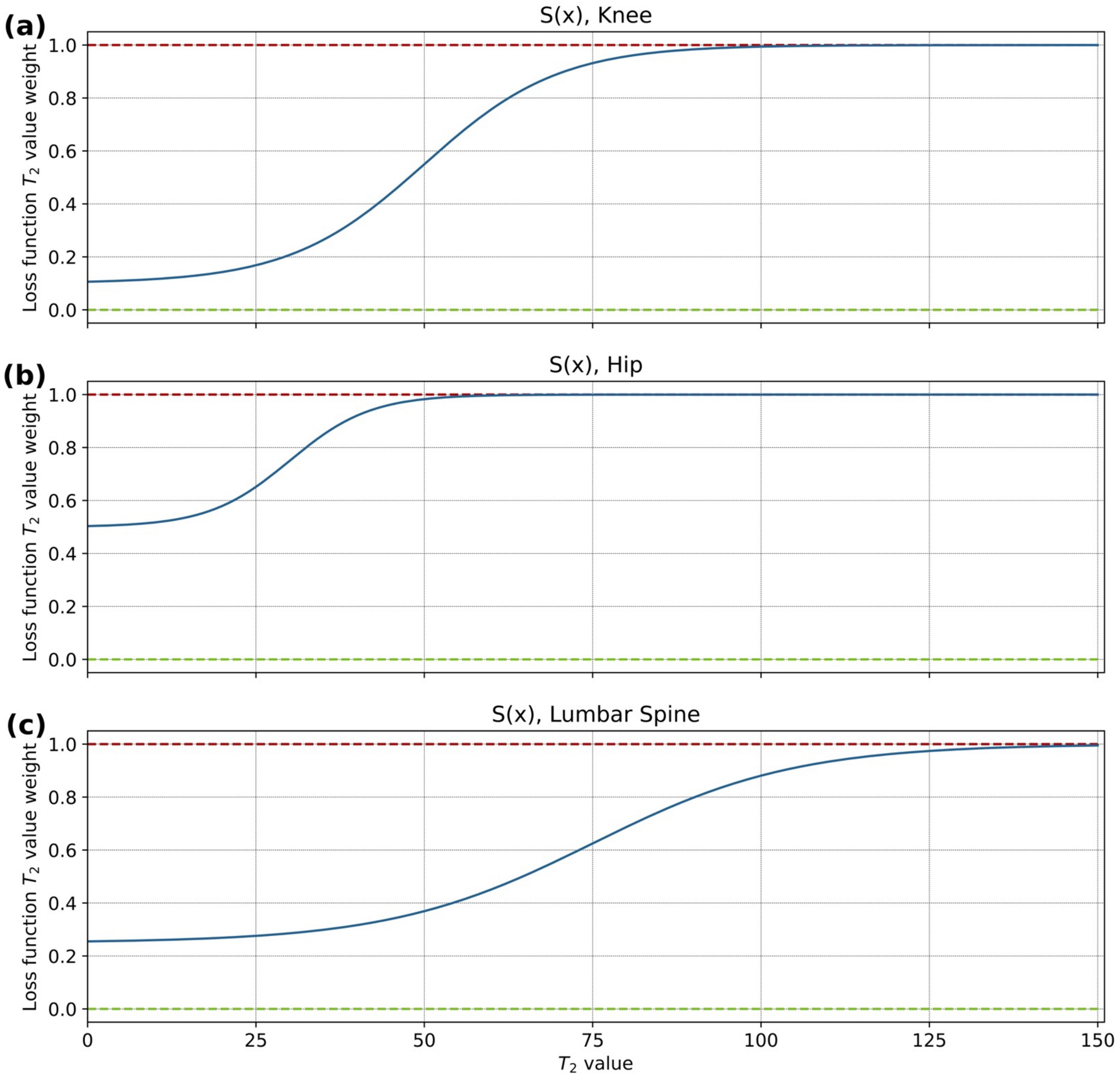

Supplementary Information Figure S2: Modified sigmoid function for knee, hip and lumbar spine pipelines. T_2 values in each architecture were fed through these sigmoid functions to determine an equivalent S(x) for the given pixel to be used for ROI-specific L_1 losses in network training. Sigmoid functions thus assign higher weight to correct prediction of higher T_2 values, which can be lost due to aliasing when undersampling images, particularly in local T_2 value elevations. Additionally, S(x) saturates signal above some threshold, allowing network training to focus on correct predictions in T_2 value ranges that are more physiologically realistic for cartilage and IVDs.

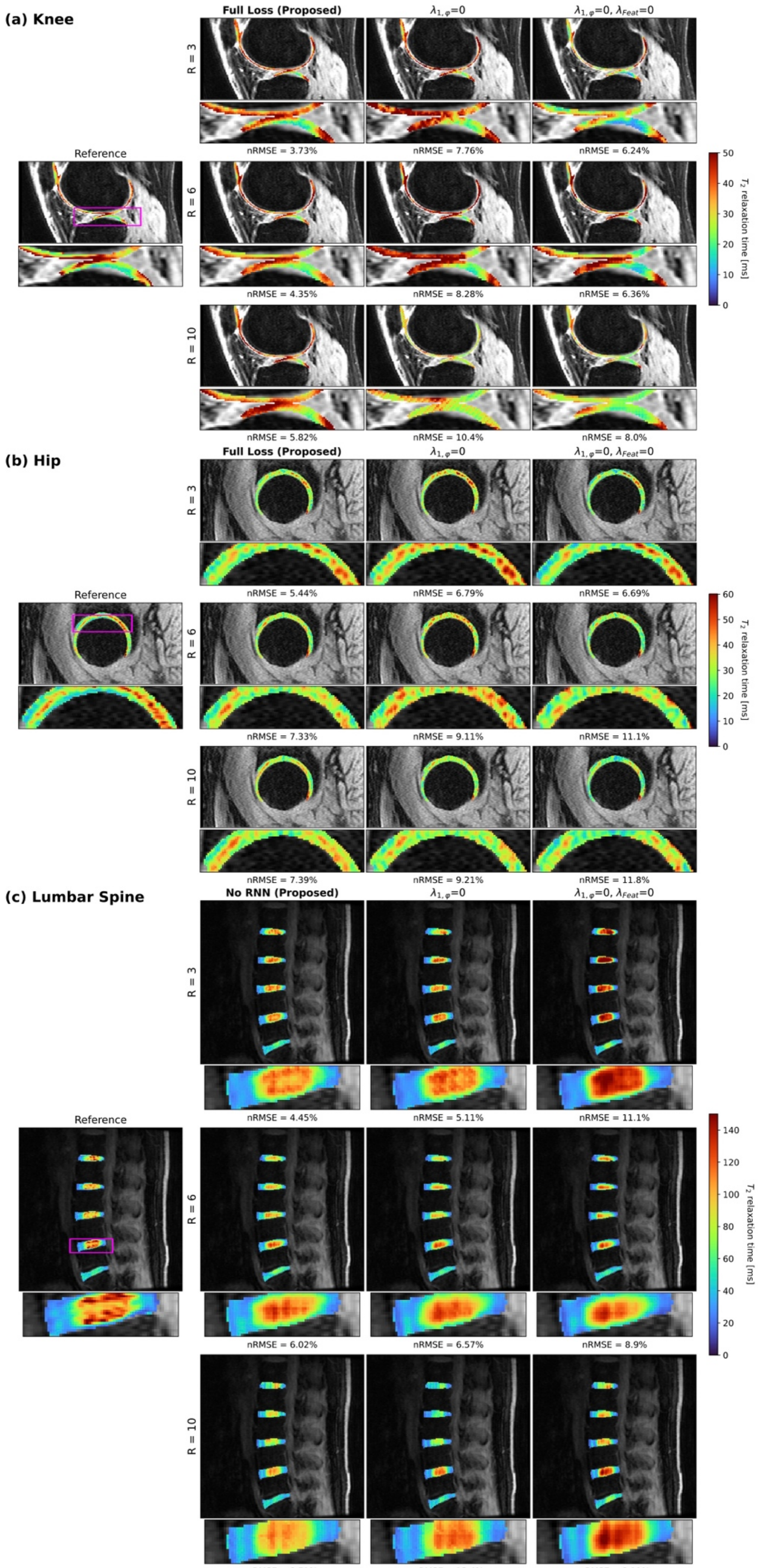

Supplementary Information Figure S3: Predicted T_2 maps for proposed models and equivalent pipelines trained without ROI-specific loss. Top-performing pipelines for knee (recurrent UNet), hip (recurrent UNet), and lumbar spine

(UNet, or "No RNN"), with corresponding versions trained with ablated loss functions. Middle column ($\lambda_{1,\phi}$=0) was trained with proposed loss function with ROI-specific component ablated (global L_1, SSIM, feature-based losses remained). Right column ($\lambda_{1,\phi}$=0, λ_{Feat}=0) with an ordinary loss function (global L_1 and SSIM). Results show that full loss pipelines have lower T_2 quantification error rates across all anatomies at R=3, 6, and 10 for visualized slices than do pipelines trained without ROI-specific loss, demonstrating its value in maintaining low errors and maintaining visual fidelity to ground truth.

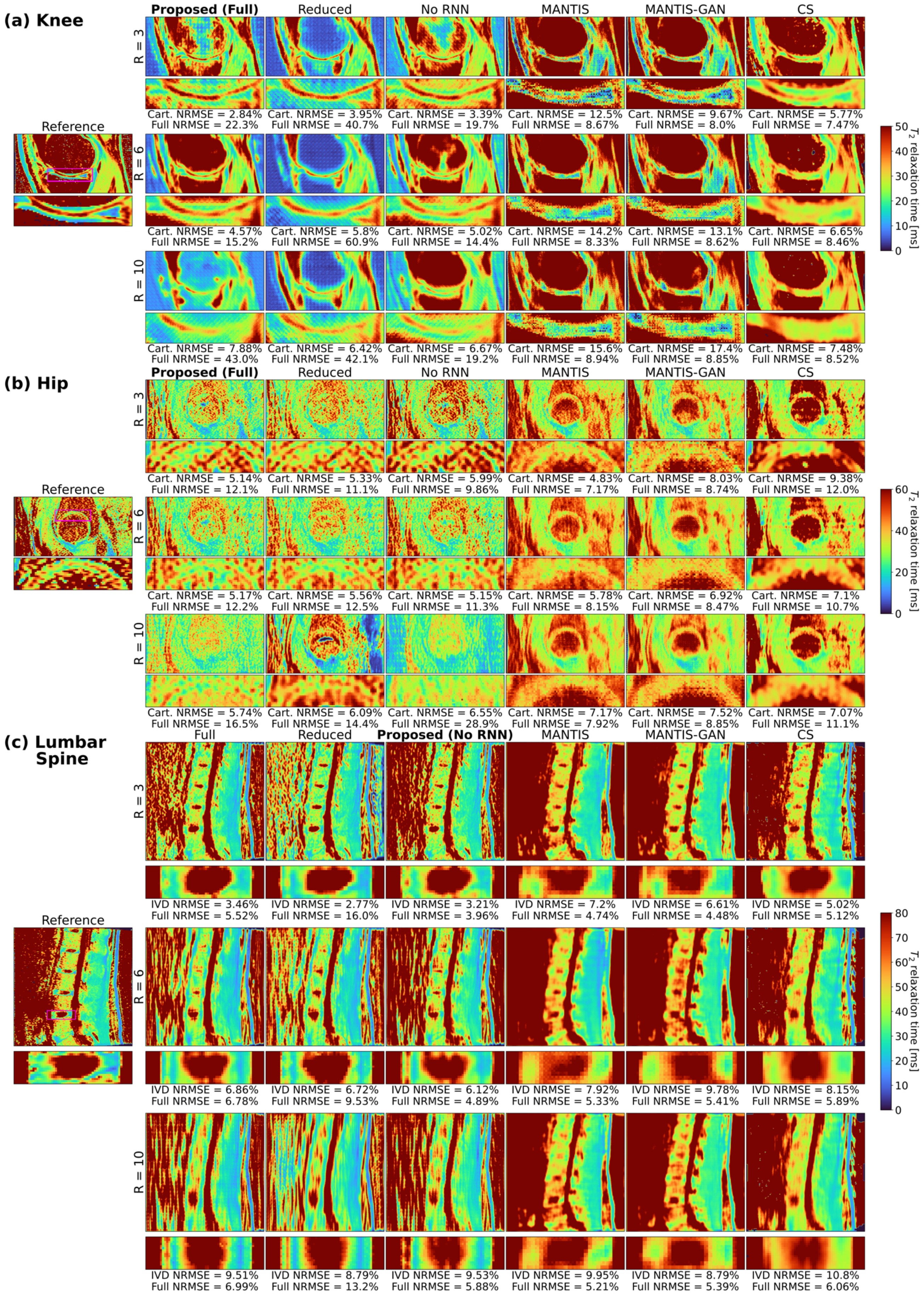

Supplementary Information Figure S4: Global T_2 value retention performance for proposed pipelines and state-of-the-art models. ROI and global T_2 quantification errors are shown for a slice within the test set for each of the knee, hip

and lumbar spine pipelines. In the knee and hip, both visually and quantitatively, T_2 maps predicted by global approaches show substantially lower global errors than do our proposed pipelines, but within cartilage ROIs, our pipelines exhibit stronger performance. These results are as expected—the ROI-specific loss function improves predictions in cartilage ROIs and degrades them globally, indicating successful training of these pipelines. In the lumbar spine, these trends are more inconsistent, possibly due to the substantially larger datasets and number of batches seen in knee and hip pipeline training as compared to the lumbar spine; some of the lumbar spine findings thus may be attributed to the randomness of training with a small dataset. Nonetheless, when afforded a sufficiently large dataset for training, the ROI-specific loss performs as expected.

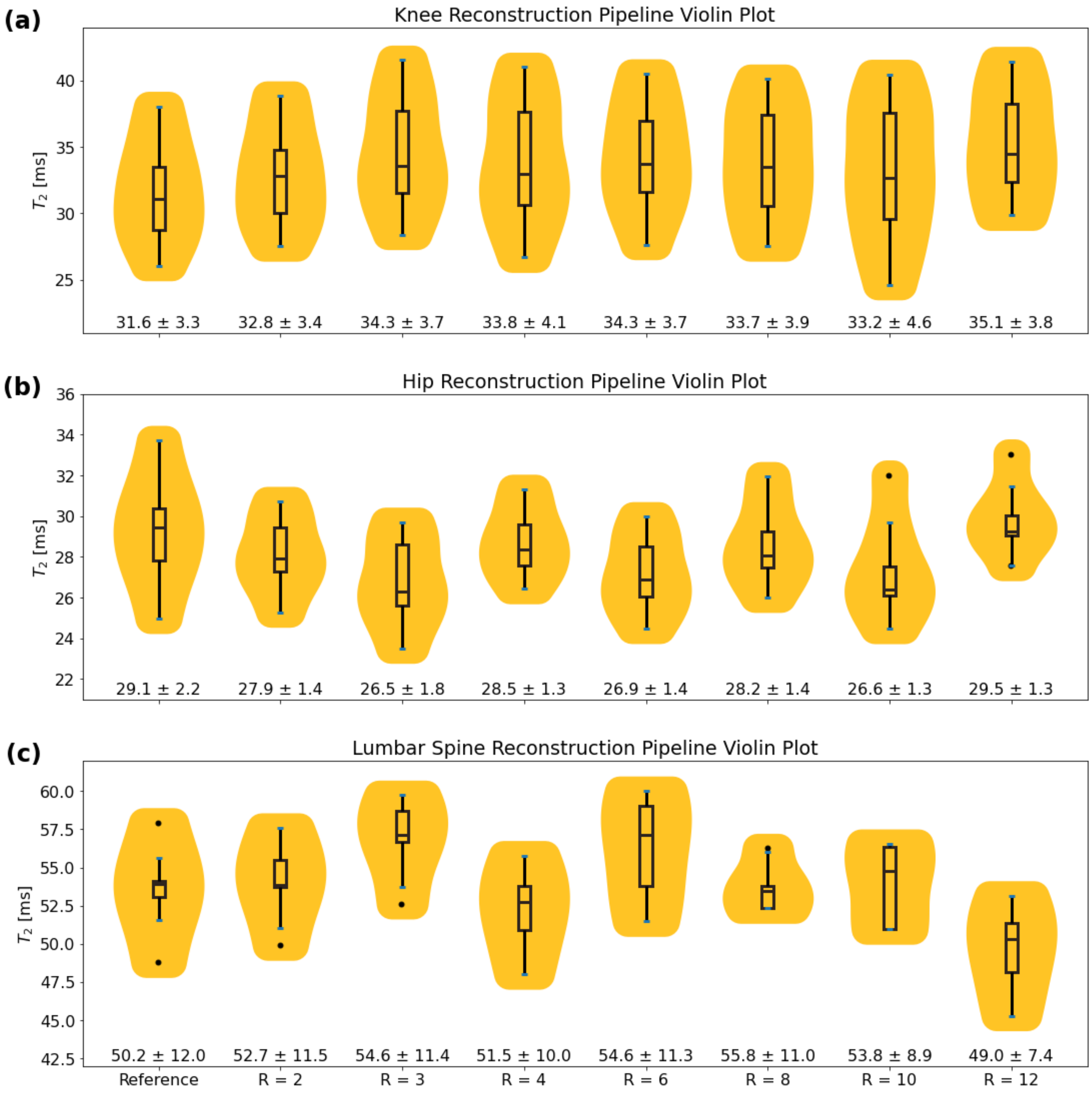

Supplementary Information Figure S5: Comparison of biases in predicted T_2 maps in knee cartilage, hip cartilage, and intervertebral discs. Violin plots of T_2 values for reference and each tested pipeline, R=2 through R=12, for **(a)** knee (n=90), **(b)** hip (n=15), and **(c)** lumbar spine (n=4). Boxplots are overlaid on violin plots, and the T_2 values are also displayed mean ±1 s.d. In conjunction with Bland-Altman plots in **Figures 3-4**, violin plots show that for knee and hip pipelines, T_2 values with are preserved with minimal bias. In the lumbar spine, while violin plots indicate some volatility in T_2 value preservation in predicted performance, bias in predicted maps was minimal at most tested R. Knee and hip pipelines thus generally maintain strong fidelity to T_2 values, whereas lumbar spine pipeline retains reasonable fidelity to T_2 values.

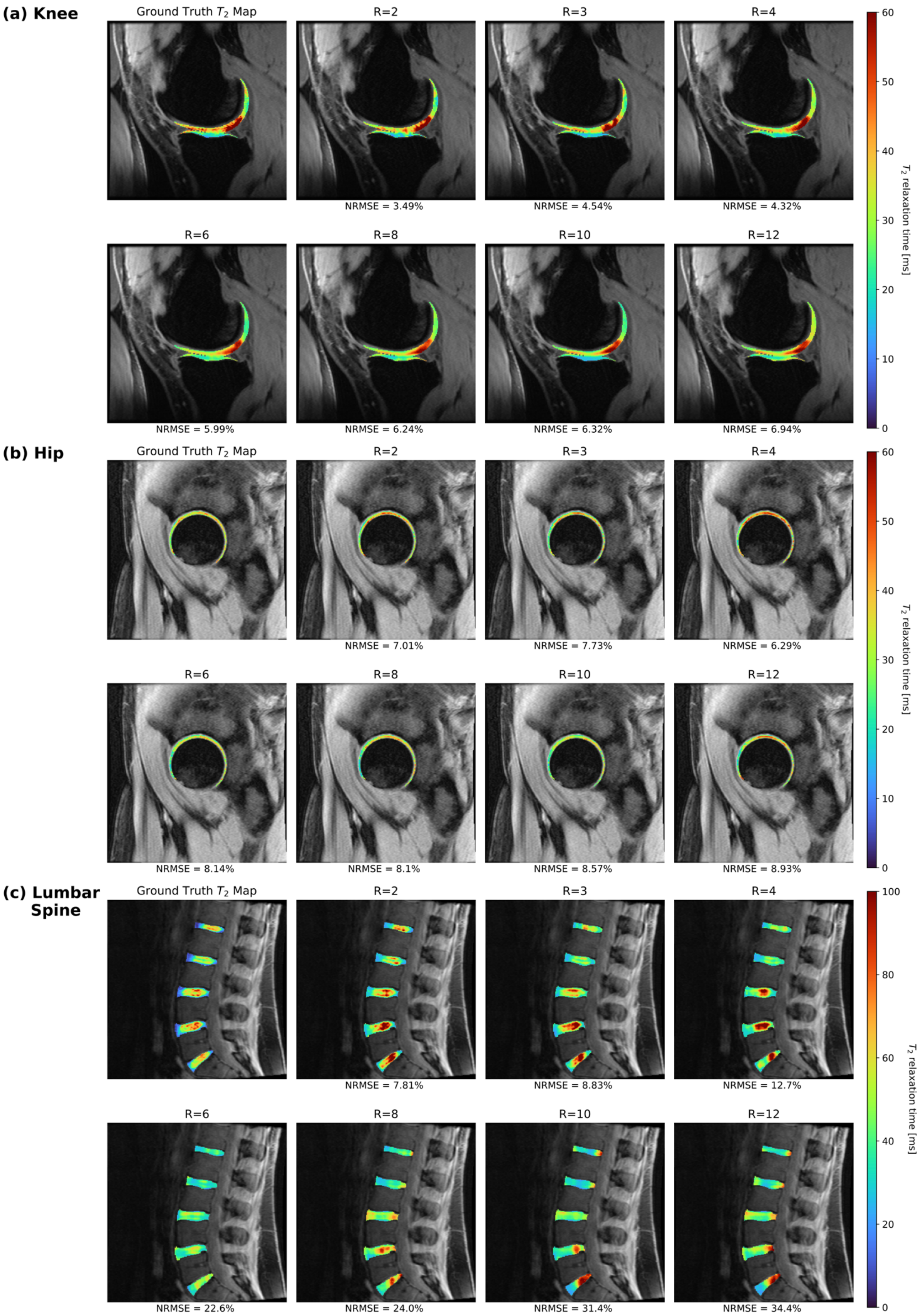

Supplementary Information Figure S6: Assessment of proposed pipeline performance on multicoil raw k-space data. MAPSS sequences were acquired for 3 knee, 2 hip, and 2 lumbar spine volunteers. Multicoil raw k-space data (after ARC

reconstruction for knee and hip) was undersampled with the same patterns applied on retrospectively undersampled coil-combined images used during training, and k-space lines were also shared as with the coil-combined approach. Resulting k-space was filtered, inverse Fourier transformed, and processed with an in-house pipeline developed to replicate all image post-processing steps normally used in generating DICOM images, thereby generated coil-combined magnitude images of multi-coil undersampled images. These coil-combined equivalents of undersampled data were fed through corresponding pipelines, yielding predicted T_2 maps. Visually, and by T_2 quantification errors, knee and hip pipelines exhibit strong performance through R=12, preserving local T_2 value elevations at most R. Slight degradation in performance was observed at lower R for hip pipelines relative to benchmarks attained on coil-combined magnitude undersampled images, but at other R for knee and hip, performance matched expectations. In the lumbar spine, performance matched expectations through R=4, declining sharply at R=6 and higher. This may be due to the smaller dataset used for lumbar spine pipeline training fewer k_z slices in lumbar spine MAPSS acquisitions that exacerbated effects of undersampling as compared to knee and hip, yielding much lower SNR in aliased model input images.

Acquisition Type	Knee	Hip	Lumbar Spine
Ground Truth	5 minutes, 53 seconds	8 minutes, 15 seconds	5 minutes, 17 seconds
R=2	2 minutes, 56 seconds	4 minutes, 8 seconds	2 minutes, 39 seconds
R=3	1 minute, 58 seconds	2 minutes, 45 seconds	1 minute, 46 seconds
R=4	1 minute, 28 seconds	2 minutes, 4 seconds	1 minute, 19 seconds
R=6	59 seconds	1 minute, 23 seconds	53 seconds
R=8	44 seconds	1 minute, 2 seconds	40 seconds
R=10	35 seconds	50 seconds	32 seconds
R=12	29 seconds	41 seconds	26 seconds

Supplementary Information Table S1: Acquisition times for MAPSS at tested R. Acquisition times for the full MAPSS sequence if the proposed undersampling patterns were implemented for $T_{1\rho}$ and T_2 preparation and image acquisition. If acquisition times were desired solely for T_2 weighted images acquired from MAPSS, all acquisition times in this table would need to be multiplied by 0.571.

Anatomy	Fold	Training			Validation			Test		
		**Patients**	**Scans**	**Slices**	**Patients**	**Scans**	**Slices**	**Patients**	**Scans**	**Slices**
Knee	1	144	265	5,591	50	91	1,952	50	90	1,928
	2	144	262	5,619	50	93	1,960	50	90	1,892
	3	144	259	5,480	50	81	1,739	50	106	2,252
Hip	1	39	59	1,533	15	15	390	13	15	390
	2	39	59	1,533	12	15	390	13	15	390
	3	40	59	1,533	13	15	390	15	15	390
Lumbar spine	1	13	14	112	4	5	42	4	5	40
	2	15	16	130	4	4	32	4	4	32
	3	14	16	130	4	4	32	4	4	32

Supplementary Information Table S2: Information for cross-validation splits in knee, hip and lumbar spine datasets. Training, validation, and test data splits of MAPSS acquisitions for all three folds, by patient and by total number of scans. In lumbar spine, total scans exceeded number of patients because some patients had spines scanned multiple times, whereas for knee and hip, both knees or both hips of some patients may have been scanned. To prevent data leakage, all scans of a particular patient was only placed into one of the three datasets. Unless otherwise specified, all results in paper are reported from fold 1 split; additional splits 2 and 3 were made to assess robustness of T_2 quantification performance and texture retention to different datasets used, as described in subsequent Supplementary information tables.

λ_{L_1}	$\lambda_{L_{1,\phi}}$	λ_{SSIM}	$\lambda_{Feat.}$	**RNN**	**Reduced Params**	**Knee**		**Hip**		**Lumbar Spine**	
						**NRMSE (%)**	**Pearson's r**	**NRMSE (%)**	**Pearson's r**	**NRMSE (%)**	**Pearson's r**
			X	X		10900 ± 1740	0.006	12100 ± 3440	-0.006	43700 ± 14800	-0.008
		X		X		17.1 ± 4.62	0.355***	24.1 ± 7.27	0.509***	26.3 ± 18.1	0.696***
		X	X	X		13.9 ± 2.05	0.432***	12.0 ± 2.38	0.468***	31.4 ± 19.8	0.676***
	X			X		12.5 ± 7.75	**0.598*****	10.8 ± 3.62	0.557***	19.0 ± 7.79	0.671***
	X		X	X		10.9 ± 6.09	0.587***	13.5 ± 8.41	0.380***	67700 ± 26700	0.002
	X	X		X		11.9 ± 7.35	**0.613*****	2800 ± 2570	0.196***	30.4 ± 24.2	0.703***
	X	X	X	X		**9.63 ± 4.92**	**0.617*****	**9.45 ± 3.0**	**0.599*****	**12.7 ± 3.75**	**0.746*****
X				X		22.9 ± 3.36	0.315**	21.9 ± 6.49	0.253***	37.2 ± 21.2	0.552***
X			X	X		14.0 ± 2.41	0.295***	16.7 ± 5.14	0.198***	37600 ± 13100	-0.003
X		X		X		17.4 ± 5.3	0.419***	12.5 ± 2.47	0.494***	13.1 ± 3.38	0.747***
X		X	X	X		14.9 ± 3.61	0.317***	12.4 ± 2.61	0.379***	59.5 ± 32.3	0.245***
X	X			X		10.1 ± 4.18	0.552***	22.6 ± 5.51	0.529***	**12.1 ± 3.86**	0.720***
X	X		X	X		11.6 ± 4.78	0.563***	19.0 ± 4.67	0.427***	24200 ± 8850	-0.008
X	X	X		X		**8.97 ± 3.6**	0.578***	11.9 ± 3.66	0.569***	13.6 ± 6.88	**0.747*****
X	X	X	X	X		**9.15 ± 2.56**	0.586***	**6.94 ± 1.94**	**0.596*****	13.3 ± 4.48	**0.742*****
X	X	X	X	X	X	**9.59 ± 3.83**	0.574***	**6.33 ± 1.33**	**0.588*****	**13.0 ± 2.63**	0.723***
X	X	X	X			10.1 ± 4.42	**0.609*****	**6.98 ± 1.45**	**0.558*****	**12.0 ± 3.07**	**0.742*****

Supplementary Information Table S3: Loss function ablation study results. Network was trained with 4 loss function components: L_1 loss across slice, L_1 loss in tissue of interest, SSIM loss, and VGG-19 feature-based loss function. Weightings of those components were λ_{L_1}, $\lambda_{L_{1,\phi}}$, λ_{SSIM}, and $\lambda_{Feat.}$, respectively. Ablation study results are shown at R=8, with "X's" in table noting that the given loss function component was not ablated in that trained pipeline version. If loss component was ablated, its λ was set to 0; if not, it remained at its value optimized through the constrained hyperparameter search. In addition, the RNN portion of the network was ablated while keeping all loss components constant, while a separate pipeline version was trained with all layer depths being half of what is depicted in **Fig. 1**. "X's" mark ablations in which the RNN was part of the network design and/or when the reduced overall number of network parameters was used in the architecture. All metrics are calculated in the tissues of interest (cartilage or IVD) and calculated at a cartilage compartment/IVD level. NRMSEs of ablated and original pipelines are presented ±1 s.d., and significance of Pearson's r is denoted as follows: * $P < 0.05$, ** $P < 0.01$, *** $P < 0.001$ (knee: n=90; hip: n=15; lumbar spine: n=5). The top 4 ablations for knee, hip and lumbar spine in NRMSE and Pearson's r are bolded. In knee and hip pipelines, all loss function components are necessary to obtain among the strongest performing models in both metrics. In the lumbar spine, substantial performance improvements are seen when the RNN is ablated; possibly, simpler network is better suited to the smaller dataset size.

λ_{L_1}	$\lambda_{L_{1,\phi}}$	λ_{SSIM}	$\lambda_{Feat.}$	**RNN**	**Reduced Params**	**Knee**	**Hip**	**Lumbar Spine**
			X	X		3440.0 ± 864.0	3540.0 ± 1060.0	20500.0 ± 10100.0
		X		X		5.4 ± 1.19	7.03 ± 2.17	12.4 ± 9.51
		X	X	X		4.39 ± 0.6	3.52 ± 0.902	14.8 ± 10.6
	X			X		3.94 ± 2.6	3.17 ± 1.18	8.92 ± 3.7
	X		X	X		3.43 ± 2.05	3.93 ± 2.92	31800.0 ± 17000.0
	X	X		X		3.77 ± 2.44	817.0 ± 823.0	14.3 ± 12.6
	X	X	X	X		3.04 ± 1.67	2.76 ± 1.07	5.94 ± 2.1
X				X		7.24 ± 1.18	6.41 ± 2.32	17.5 ± 11.9
X			X	X		4.43 ± 0.601	4.87 ± 1.79	17700.0 ± 8830.0
X		X		X		5.49 ± 1.3	3.66 ± 0.782	6.17 ± 0.821
X		X	X	X		4.7 ± 0.811	3.63 ± 0.676	27.9 ± 18.4
X	X			X		3.2 ± 1.39	6.59 ± 2.02	5.68 ± 1.43
X	X		X	X		3.67 ± 1.54	5.55 ± 1.65	11400.0 ± 5910.0
X	X	X		X		2.84 ± 1.21	3.46 ± 1.31	6.37 ± 3.59
X	X	X	X	X		2.89 ± 0.906	2.03 ± 0.668	6.24 ± 2.46
X	X	X	X	X	X	3.03 ± 1.29	1.85 ± 0.418	6.11 ± 1.15
X	X	X	X			3.21 ± 1.52	2.04 ± 0.488	5.62 ± 1.26

Supplementary Information Table S4: Loss function ablation study T_2 value mean errors. Additional details on loss function ablation study. NRMSE was calculated across all cartilage or IVD on a patient level for all ablated models, and these NRMSEs were multiplied by corresponding mean reference T_2 values to convert T_2 quantification error rates into T_2 estimation errors. Estimation errors are reported mean ±1 s.d. [ms] (knee: n=90; hip: n=15; lumbar spine: n=5).

Tissue	R	Tissue	Proposed Model	$\lambda_{1,\phi}$=0	$\lambda_{1,\phi}$=0, λ_{Feat}=0	MANTIS	MANTIS-GAN	CS
Knee	2	Cartilage	0.748***	0.658***	0.685***	0.587***	0.611***	0.620***
		Global	0.667***	0.675***	0.677***	0.700***	0.703***	0.581***
	3	Cartilage	0.695***	0.491***	0.573***	0.467***	0.502***	0.559***
		Global	0.383***	0.618***	0.683***	0.681***	0.693***	0.566***
	4	Cartilage	0.651***	0.376***	0.558***	0.451***	0.467***	0.486***
		Global	0.149***	0.575***	0.693***	0.672***	0.670***	0.554***
	6	Cartilage	0.612***	0.465***	0.442***	0.397***	0.378*	0.445***
		Global	0.487***	0.663***	0.687***	0.667***	0.659***	0.547***
	8	Cartilage	0.585***	0.383***	0.450***	0.352***	0.364**	0.410***
		Global	0.440***	0.661***	0.672***	0.659***	0.654***	0.548***
	10	Cartilage	0.555***	0.124**	0.346***	0.327***	0.333***	0.386***
		Global	0.165***	0.540***	0.623***	0.651***	0.653***	0.552***
	12	Cartilage	0.491***	0.339***	0.396***	0.290***	0.287***	0.381***
		Global	0.559***	0.613***	0.630***	0.659***	0.656***	0.557***
Hip	2	Cartilage	0.794***	0.562***	0.655***	0.716***	0.514***	0.310***
		Global	0.683***	0.713***	0.727***	0.755***	0.699***	0.534***
	3	Cartilage	0.705***	0.541***	0.612***	0.596***	0.372***	0.332***
		Global	0.607***	0.669***	0.660***	0.723***	0.659***	0.549***
	4	Cartilage	0.646***	0.459***	0.540***	0.510***	0.333***	0.339***
		Global	0.609***	0.594***	0.610***	0.707***	0.641***	0.562***
	6	Cartilage	0.587***	0.458***	0.480***	0.382***	0.321***	0.334***
		Global	0.555***	0.595***	0.643***	0.690***	0.634***	0.573***
	8	Cartilage	0.598***	0.344***	0.437***	0.334***	0.237***	0.347***
		Global	0.630***	0.577***	0.627***	0.684***	0.660***	0.574***
	10	Cartilage	0.558***	0.355***	0.434***	0.279***	0.268***	0.335***
		Global	0.412***	0.429***	0.551***	0.686***	0.615***	0.569***
	12	Cartilage	0.517***	0.427***	0.423***	0.280***	0.228***	0.349***
		Global	0.619***	0.625***	0.622***	0.682***	0.622***	0.578***
Lumbar Spine	2	IVDs	0.884***	0.850***	0.879***	0.784***	0.785***	0.802***
		Global	0.836***	0.673***	0.772***	0.816***	0.821***	0.812***
	3	IVDs	0.832***	0.823***	0.846***	0.717***	0.712***	0.777***
		Global	0.797***	0.707***	0.711***	0.784***	0.786***	0.788***
	4	IVDs	0.819***	0.804***	0.827***	0.680***	0.671***	0.723***
		Global	0.783***	0.696***	0.743***	0.771***	0.774***	0.772***
	6	IVDs	0.771***	0.764***	0.761***	0.660***	0.658***	0.728***
		Global	0.766***	0.737***	0.712***	0.764***	0.770***	0.760***
	8	IVDs	0.742***	0.245***	0.747***	0.631***	0.645***	0.695***
		Global	0.749***	0.664***	0.720***	0.756***	0.757***	0.752***
	10	IVDs	0.672***	0.651***	0.698***	0.647***	0.636***	0.648***
		Global	0.728***	0.707***	0.669***	0.762***	0.761***	0.747***
	12	IVDs	0.643***	0.581***	0.654***	0.651***	0.614***	0.586***
		Global	0.707***	0.661***	0.686***	0.760***	0.762***	0.746***

Supplementary Information Table S5: ROI and global correlations between predicted and ground truth T_2 maps. Pearson's r between predicted and ground truth T_2 maps for proposed model trained with full 4-component loss function,

proposed model trained with just the ROI-specific loss component ablated ($\lambda_{1,\phi}$=0), and proposed model trained with an ordinary loss function of L_1 and SSIM ($\lambda_{1,\phi}$=0, λ_{Feat}=0) as part of ablation study. Correlations are also provided between predictions and ground truth for 3 state-of-the-art models. Significance of Pearson's r is denoted as follows: * $P < 0.05$, ** $P < 0.01$, *** $P < 0.001$ (knee: n=90; hip: n=15; lumbar spine: n=5). For any given R, the strongest correlation within the ROI is highlighted in red, whereas the strongest correlation globally is highlighted in blue. Across the hip and knee pipelines, each of which had large datasets available for training, correlations are strongest within cartilage ROIs for the proposed pipelines across all R, while for all R, state-of-the-art DL pipelines (MANTIS, MANTIS-GAN) exhibited stronger correlations globally to ground truth. Similarly, when the ROI-specific loss function was ablated, for nearly all tested R in hip and knee, correlations became stronger globally than for the proposed pipelines. This is indicative of successful training and the role of the ROI-specific loss function: with a sufficiently large training set, it improves results within cartilage ROIs at the expense of global performance, allowing for ROI-specific model optimization. These trends were inconsistent inn the lumbar spine, likely owing to the very small dataset size that added randomness to the training process; some results thus may be a result of more complete training rather than the specific utility of the ROI-specific loss. If trained with a larger dataset, the lumbar spine results would be expected to mirror those observed in the knee and hip.

Tissue	R	Full Model	Reduced Parameters	No RNN	MANTIS	MANTIS-GAN	CS
Knee Cartilage	2	1.75 ± 0.449	1.92 ± 1.1	1.5 ± 0.62	4.56 ± 0.631	4.28 ± 0.726	2.84 ± 0.951
	3	2.06 ± 0.773	2.27 ± 1.07	2.02 ± 0.884	5.23 ± 0.694	4.79 ± 0.652	3.15 ± 1.08
	4	2.38 ± 1.01	3.02 ± 1.81	2.39 ± 1.08	5.24 ± 0.869	4.95 ± 1.03	3.74 ± 1.26
	6	2.56 ± 0.933	3.38 ± 2.18	2.67 ± 1.21	4.82 ± 0.709	5.16 ± 0.903	3.93 ± 1.44
	8	2.82 ± 0.93	3.03 ± 1.29	3.21 ± 1.52	5.28 ± 0.673	5.46 ± 0.689	4.1 ± 1.41
	10	3.09 ± 1.14	3.21 ± 1.26	2.95 ± 1.24	5.55 ± 0.654	5.29 ± 0.822	4.26 ± 1.38
	12	3.37 ± 0.822	3.14 ± 1.3	3.31 ± 1.21	5.77 ± 1.0	6.48 ± 1.22	4.27 ± 1.46
Hip Cartilage	2	1.16 ± 0.34	1.2 ± 0.349	1.11 ± 0.229	1.34 ± 0.287	2.4 ± 0.327	4.31 ± 0.745
	3	1.91 ± 0.516	1.64 ± 0.519	1.54 ± 0.332	1.87 ± 0.363	2.93 ± 0.382	3.74 ± 0.814
	4	1.8 ± 0.348	1.8 ± 0.525	1.71 ± 0.273	2.14 ± 0.465	2.91 ± 0.467	3.42 ± 0.593
	6	2.37 ± 0.598	2.4 ± 0.676	2.19 ± 0.484	2.52 ± 0.643	2.83 ± 0.497	3.43 ± 0.559
	8	2.04 ± 0.676	1.85 ± 0.418	2.04 ± 0.488	2.98 ± 0.789	3.52 ± 0.767	3.04 ± 0.681
	10	2.63 ± 0.873	2.37 ± 0.4	2.54 ± 1.22	2.85 ± 0.637	3.05 ± 0.563	2.96 ± 0.685
	12	2.26 ± 0.499	2.42 ± 0.737	2.14 ± 0.433	2.85 ± 0.614	3.35 ± 0.685	3.0 ± 0.748
Lumbar Spine IVDs	2	3.15 ± 0.602	3.22 ± 0.571	2.28 ± 0.4	4.12 ± 1.06	4.2 ± 1.06	4.74 ± 1.93
	3	4.66 ± 0.854	4.11 ± 0.434	3.35 ± 0.478	5.17 ± 0.931	5.29 ± 1.14	4.45 ± 1.14
	4	4.85 ± 0.736	4.57 ± 0.859	3.48 ± 0.473	5.7 ± 1.17	5.91 ± 1.29	5.3 ± 1.65
	6	5.68 ± 1.74	5.75 ± 1.24	4.84 ± 1.3	7.32 ± 2.28	5.7 ± 1.07	5.62 ± 1.63
	8	6.28 ± 0.967	6.11 ± 1.15	5.62 ± 1.26	6.22 ± 1.27	5.96 ± 0.983	6.02 ± 1.53
	10	7.18 ± 0.725	6.95 ± 0.705	6.93 ± 1.1	6.48 ± 1.56	6.19 ± 1.29	7.04 ± 2.35
	12	8.48 ± 1.18	10.8 ± 2.82	8.84 ± 2.24	6.94 ± 2.18	6.62 ± 1.53	11.7 ± 7.04

Supplementary Information Table S6: T_2 value equivalents of quantification errors in cartilage, IVDs across all models. Additional details on model performance from R=2 through R=12 within cartilage compartments and at disc levels. As in Supplementary Information Table S4**,** NRMSE was calculated across cartilage or IVD compartment on a patient level for all ablated models, and these NRMSEs were multiplied by corresponding mean reference T2 values to convert quantification error rates into T2 estimation errors. Estimation errors are reported mean ±1 s.d. [ms] (knee: n=90; hip: n=15; lumbar spine: n=5).

R	Tissue	Full Model	Reduced Parameters	No RNN	MANTIS	MANTIS-GAN	CS
2	Lateral Femoral Condyle	5.76 ± 2.63	8.95 ± 10.7	**5.67 ± 4.72**	13.9 ± 4.21	13.1 ± 4.29	11.5 ± 9.38
	Lateral Tibial Condyle	6.02 ± 2.35	6.9 ± 4.56	**5.49 ± 3.72**	17.6 ± 7.43	17.9 ± 6.71	9.56 ± 6.7
	Medial Femoral Condyle	5.31 ± 2.06	4.54 ± 1.75	**4.18 ± 1.69**	15.2 ± 6.93	14.2 ± 6.71	12.2 ± 8.76
	Medial Tibial Condyle	8.1 ± 4.94	10.1 ± 12.4	**7.73 ± 6.48**	22.5 ± 7.98	20.6 ± 6.68	14.9 ± 7.87
	Trochlear	7.19 ± 4.45	6.56 ± 5.32	**5.14 ± 2.7**	16.6 ± 5.3	17.1 ± 8.73	5.23 ± 3.26
	Patellar	4.08 ± 1.24	4.69 ± 1.55	**3.52 ± 1.38**	12.3 ± 2.89	11.2 ± 3.95	4.02 ± 2.15
	All Cartilage	5.52 ± 1.25	6.07 ± 3.21	**4.76 ± 1.78**	14.4 ± 2.85	13.5 ± 3.3	8.92 ± 3.2
3	Lateral Femoral Condyle	**7.37 ± 5.35**	9.74 ± 8.88	8.25 ± 7.43	16.3 ± 4.71	14.9 ± 5.24	14.4 ± 10.4
	Lateral Tibial Condyle	8.01 ± 3.83	7.99 ± 5.36	**7.41 ± 4.7**	18.8 ± 5.91	17.1 ± 6.03	10.1 ± 4.98
	Medial Femoral Condyle	**5.77 ± 2.19**	6.46 ± 2.79	6.33 ± 2.74	17.5 ± 3.18	15.6 ± 3.79	13.6 ± 7.82
	Medial Tibial Condyle	10.6 ± 8.22	10.9 ± 11.4	**10.2 ± 9.52**	22.9 ± 7.21	19.9 ± 7.21	16.1 ± 8.27
	Trochlear	7.13 ± 4.43	7.55 ± 5.3	**6.71 ± 4.26**	20.4 ± 8.83	18.9 ± 8.3	6.02 ± 2.4
	Patellar	5.03 ± 1.39	5.29 ± 1.49	**4.32 ± 1.79**	14.1 ± 4.94	12.9 ± 3.81	4.65 ± 2.18
	All Cartilage	6.52 ± 2.17	7.18 ± 3.08	**6.39 ± 2.59**	16.5 ± 3.43	15.1 ± 2.89	9.92 ± 3.23
4	Lateral Femoral Condyle	**10.7 ± 10.1**	17.4 ± 20.8	**10.7 ± 9.41**	17.2 ± 5.44	15.6 ± 6.26	15.1 ± 10.5
	Lateral Tibial Condyle	9.2 ± 4.64	12.3 ± 7.56	**8.98 ± 5.28**	20.5 ± 6.87	18.9 ± 5.65	12.7 ± 7.0
	Medial Femoral Condyle	**7.16 ± 2.85**	11.8 ± 5.28	8.44 ± 4.67	17.7 ± 5.0	17.9 ± 4.71	16.1 ± 8.94
	Medial Tibial Condyle	**11.3 ± 10.2**	16.6 ± 17.7	12.0 ± 11.2	23.6 ± 7.58	21.9 ± 6.81	19.0 ± 9.19
	Trochlear	7.4 ± 4.97	8.88 ± 8.47	**7.03 ± 5.1**	19.3 ± 10.3	21.7 ± 17.6	8.15 ± 2.97
	Patellar	5.11 ± 1.26	**4.61 ± 1.73**	4.76 ± 1.99	13.3 ± 3.5	12.3 ± 4.45	4.86 ± 1.79
	All Cartilage	**7.54 ± 2.96**	9.56 ± 5.47	7.56 ± 3.19	16.6 ± 3.73	15.7 ± 4.5	11.8 ± 3.73
6	Lateral Femoral Condyle	**10.9 ± 8.25**	20.1 ± 25.0	11.9 ± 11.0	14.9 ± 4.08	16.8 ± 5.3	16.0 ± 11.2
	Lateral Tibial Condyle	**10.3 ± 4.75**	15.7 ± 9.91	**10.3 ± 6.11**	21.6 ± 7.29	19.6 ± 4.92	14.2 ± 7.92
	Medial Femoral Condyle	**7.93 ± 3.36**	11.2 ± 6.09	9.76 ± 5.98	16.6 ± 5.09	20.1 ± 8.09	17.2 ± 8.43
	Medial Tibial Condyle	**12.4 ± 8.32**	17.3 ± 20.1	13.0 ± 10.9	23.3 ± 5.51	22.5 ± 5.66	19.3 ± 9.17
	Trochlear	**7.0 ± 4.39**	10.4 ± 11.5	7.43 ± 4.81	17.6 ± 9.21	19.5 ± 10.5	9.7 ± 3.28
	Patellar	5.69 ± 1.29	5.45 ± 2.11	**5.45 ± 1.83**	12.0 ± 3.1	12.4 ± 3.5	5.76 ± 2.33
	All Cartilage	**8.09 ± 2.65**	10.7 ± 6.67	8.44 ± 3.49	15.2 ± 2.33	16.3 ± 3.91	12.4 ± 4.1
8	Lateral Femoral Condyle	**11.6 ± 9.1**	14.2 ± 14.6	14.7 ± 13.6	16.2 ± 4.83	16.4 ± 4.89	17.2 ± 10.3
	Lateral Tibial Condyle	**10.4 ± 4.13**	11.0 ± 5.98	11.9 ± 6.75	20.4 ± 5.84	19.3 ± 5.03	14.8 ± 7.17
	Medial Femoral Condyle	**9.5 ± 5.11**	12.8 ± 4.97	14.2 ± 8.74	18.3 ± 5.42	17.2 ± 4.03	17.5 ± 8.66
	Medial Tibial Condyle	**12.9 ± 8.83**	14.5 ± 10.8	15.8 ± 14.0	23.2 ± 5.11	23.1 ± 6.36	19.3 ± 8.85
	Trochlear	8.49 ± 4.77	8.64 ± 6.49	**8.38 ± 5.54**	17.3 ± 4.21	18.7 ± 4.49	10.7 ± 3.58
	Patellar	**6.33 ± 1.24**	6.34 ± 1.6	5.68 ± 2.18	13.6 ± 3.46	14.8 ± 3.29	6.05 ± 2.35
	All Cartilage	**8.94 ± 2.66**	9.59 ± 3.83	10.1 ± 4.42	16.7 ± 2.73	17.3 ± 2.39	12.9 ± 3.93
10	Lateral Femoral Condyle	13.2 ± 10.9	13.4 ± 10.9	**12.5 ± 8.94**	16.7 ± 4.32	18.1 ± 6.8	16.6 ± 9.32
	Lateral Tibial Condyle	**11.5 ± 5.28**	13.7 ± 5.03	11.7 ± 6.06	21.0 ± 4.54	20.0 ± 4.26	14.8 ± 6.67
	Medial Femoral Condyle	12.2 ± 5.9	12.0 ± 6.17	**11.3 ± 6.13**	18.7 ± 5.24	21.4 ± 8.17	17.0 ± 7.71
	Medial Tibial Condyle	17.4 ± 14.3	**14.4 ± 9.27**	15.0 ± 11.8	23.9 ± 6.07	22.7 ± 6.22	20.0 ± 8.92
	Trochlear	8.88 ± 6.27	8.88 ± 5.54	**8.25 ± 5.51**	18.9 ± 6.22	19.0 ± 9.7	12.2 ± 4.15
	Patellar	6.31 ± 1.67	6.47 ± 1.52	**5.54 ± 1.96**	14.9 ± 3.48	11.6 ± 3.25	6.8 ± 2.72
	All Cartilage	9.77 ± 3.44	10.2 ± 3.61	**9.35 ± 3.5**	17.6 ± 2.48	16.7 ± 3.44	13.4 ± 3.76
12	Lateral Femoral Condyle	**13.0 ± 7.39**	13.8 ± 12.4	13.6 ± 7.85	17.7 ± 5.29	21.2 ± 7.46	16.7 ± 9.75
	Lateral Tibial Condyle	13.3 ± 4.03	**12.3 ± 5.86**	13.7 ± 5.92	22.7 ± 6.26	23.9 ± 6.26	15.0 ± 6.26
	Medial Femoral Condyle	**12.1 ± 5.68**	12.6 ± 6.34	14.0 ± 9.33	22.4 ± 8.65	28.3 ± 10.6	17.5 ± 8.03
	Medial Tibial Condyle	15.0 ± 7.12	**14.0 ± 9.78**	15.3 ± 8.63	24.2 ± 5.75	26.2 ± 5.02	19.9 ± 8.33
	Trochlear	8.6 ± 4.45	8.2 ± 5.31	**8.18 ± 4.79**	19.8 ± 9.12	23.3 ± 11.6	12.3 ± 4.22
	Patellar	8.18 ± 1.95	6.7 ± 1.66	**6.29 ± 2.19**	14.5 ± 6.52	15.5 ± 6.5	7.09 ± 2.73
	All Cartilage	10.7 ± 2.32	**9.93 ± 3.76**	10.5 ± 3.37	18.2 ± 4.5	20.5 ± 5.58	13.4 ± 3.96

Supplementary Information Table S7: T_2 quantification errors in knee cartilage compartments for 6 tested models. Performances of all methods – our ROI-specific loss approaches, other DL and DL/model-based approaches, and a CS

approach in predicting T_2 maps in knee cartilage. NRMSEs are reported ±1 s.d., with the top performing model in each cartilage compartment at a given R shown in bold (n=16). Top performing pipelines were all pipelines with ROI-specific loss functions used in training, particularly with our full pipeline and its no RNN version being strongest. In the lateral and medial femoral condyles, T_2 quantification performance was below clinically significant thresholds for all tested R of the full pipeline, and for nearly all tested R for the no RNN pipeline.

R	Tissue	Full Model	Reduced Parameters	No RNN	MANTIS	MANTIS-GAN	CS
2	Lateral Femoral Condyle	0.712***	0.678***	**0.779*** **	0.522***	0.558***	0.555**
	Lateral Tibial Condyle	0.801***	0.775***	**0.829*** **	0.573***	0.586***	0.689***
	Medial Femoral Condyle	0.759***	0.775***	**0.826*** **	0.548***	0.605***	0.565*
	Medial Tibial Condyle	0.721***	0.697***	**0.760*** **	0.460**	0.510***	0.503***
	Trochlear	0.780***	0.762***	**0.826*** **	0.692***	0.700***	0.811***
	Patellar	0.671***	0.686***	**0.764*** **	0.527***	0.557***	0.694***
	All Cartilage	0.748***	0.736***	**0.807*** **	0.587***	0.611***	0.620***
3	Lateral Femoral Condyle	0.655***	0.562***	**0.660*** **	0.404***	0.469***	0.447*
	Lateral Tibial Condyle	0.715***	0.720***	**0.738*** **	0.416*	0.463*	0.627***
	Medial Femoral Condyle	0.723***	0.701***	**0.748*** **	0.435***	0.509***	0.505***
	Medial Tibial Condyle	0.655*	0.643**	**0.675** **	0.267	0.330	0.408*
	Trochlear	0.711***	0.715***	0.759***	0.638***	0.664***	**0.773*** **
	Patellar	0.618***	0.584***	**0.679*** **	0.363***	0.391***	0.633***
	All Cartilage	0.695***	0.668***	**0.722*** **	0.467***	0.502***	0.559***
4	Lateral Femoral Condyle	0.528***	0.512*	**0.572*** **	0.353***	0.407***	0.421**
	Lateral Tibial Condyle	0.682***	0.670***	**0.705*** **	0.368*	0.373*	0.538*
	Medial Femoral Condyle	0.666***	0.638***	**0.674*** **	0.402*	0.443***	0.463**
	Medial Tibial Condyle	0.600*	0.586***	**0.616* **	0.199*	0.218	0.319**
	Trochlear	0.710***	0.708***	**0.750*** **	0.636***	0.638***	0.700***
	Patellar	0.627***	0.629***	**0.645*** **	0.362*	0.377*	0.597***
	All Cartilage	0.651***	0.637***	**0.677*** **	0.451***	0.467***	0.486***
6	Lateral Femoral Condyle	0.475***	0.481***	**0.527*** **	0.306***	0.309***	0.349
	Lateral Tibial Condyle	0.600**	0.652***	**0.646*** **	0.245*	0.246*	0.462
	Medial Femoral Condyle	0.606***	0.629***	**0.627*** **	0.339*	0.366***	0.394
	Medial Tibial Condyle	0.530**	0.558***	**0.562* **	0.130	0.082	0.219*
	Trochlear	**0.728*** **	0.672***	0.715***	0.619***	0.599***	0.656***
	Patellar	0.557***	**0.568*** **	0.561***	0.308***	0.247***	0.527***
	All Cartilage	0.612***	0.610***	**0.629*** **	0.397***	0.378*	0.445***
8	Lateral Femoral Condyle	0.473***	0.445**	**0.486*** **	0.271*	0.278	0.285
	Lateral Tibial Condyle	0.601***	0.614**	**0.648*** **	0.216***	0.237*	0.423*
	Medial Femoral Condyle	**0.590*** **	0.558***	0.586***	0.308	0.330***	0.377*
	Medial Tibial Condyle	0.502*	0.520**	**0.536** **	0.058*	0.034	0.177*
	Trochlear	0.691***	0.673***	**0.695*** **	0.585***	0.586***	0.625***
	Patellar	0.554***	**0.564*** **	0.549***	0.270***	0.255***	0.482***
	All Cartilage	0.585***	0.574***	**0.609*** **	0.352***	0.364**	0.410***
10	Lateral Femoral Condyle	0.399	0.366***	**0.405*** **	0.242	0.275*	0.287
	Lateral Tibial Condyle	0.548*	0.504***	**0.568*** **	0.185*	0.174*	0.406*
	Medial Femoral Condyle	0.509***	0.480***	**0.511*** **	0.280*	0.347***	0.362*
	Medial Tibial Condyle	0.427***	0.433**	**0.465* **	0.016	-0.008	0.132
	Trochlear	**0.650*** **	0.628***	**0.650*** **	0.564***	0.561***	0.590***
	Patellar	**0.537*** **	0.443***	0.527***	0.203	0.285***	0.414***
	All Cartilage	0.555***	0.514***	**0.565*** **	0.327***	0.333***	0.386***
12	Lateral Femoral Condyle	0.359*	0.409*	**0.377* **	0.214	0.198	0.266
	Lateral Tibial Condyle	0.470***	**0.575*** **	0.494***	0.174*	0.184**	0.375*
	Medial Femoral Condyle	0.444***	**0.493*** **	0.435***	0.249*	0.244*	0.344
	Medial Tibial Condyle	0.339*	**0.467*** **	0.360*	-0.001	-0.022	0.090
	Trochlear	0.656***	**0.679*** **	0.672***	0.520***	0.501***	0.582***
	Patellar	0.434***	**0.512*** **	0.465***	0.157**	0.153***	0.392**
	All Cartilage	0.491***	**0.545*** **	0.511***	0.290***	0.287***	0.381***

Supplementary Information Table S8: Correlation between predicted and ground truth T_2 in knee cartilage compartments for all tested pipelines. Pearson's r between predicted and ground truth T_2 maps in knee cartilage, the

significance of which is noted as follows: * $P < 0.05$, ** $P < 0.01$, *** $P < 0.001$ (n=16). The top performing model in each cartilage compartment at each R is shown in bold. The no RNN pipeline is the best across most cartilage compartments and R, but the pipelines with ROI-specific losses substantially outperform their counterparts in most cases, exhibiting strong map performance at high R.

R	Tissue	Full Model	Reduced Parameters	No RNN	MANTIS	MANTIS-GAN	CS
2	Femoral	3.69 ± 1.0	3.94 ± 1.05	**3.33 ± 0.65**	3.85 ± 0.746	7.05 ± 1.09	9.56 ± 2.82
	Acetabular	4.4 ± 1.46	**4.37 ± 1.72**	4.54 ± 1.52	5.8 ± 1.92	9.89 ± 2.99	18.9 ± 5.8
	All Cartilage	3.97 ± 1.03	4.1 ± 1.1	**3.79 ± 0.807**	4.58 ± 0.993	8.21 ± 1.42	14.8 ± 2.78
3	Femoral	6.14 ± 1.61	5.54 ± 1.71	**4.72 ± 1.07**	5.55 ± 0.94	9.06 ± 1.21	8.56 ± 2.21
	Acetabular	7.16 ± 2.31	5.78 ± 2.44	**6.15 ± 2.07**	7.8 ± 2.37	11.3 ± 2.95	16.5 ± 5.75
	All Cartilage	6.53 ± 1.63	5.63 ± 1.68	**5.25 ± 1.13**	6.41 ± 1.31	10.0 ± 1.57	12.9 ± 3.15
4	Femoral	5.66 ± 1.01	5.81 ± 1.53	**5.23 ± 0.8**	6.27 ± 1.23	9.09 ± 1.68	8.78 ± 2.94
	Acetabular	7.0 ± 1.76	6.84 ± 2.06	**6.79 ± 1.55**	8.88 ± 3.0	11.1 ± 3.23	14.5 ± 3.76
	All Cartilage	6.15 ± 1.01	6.17 ± 1.47	**5.84 ± 0.891**	7.33 ± 1.67	9.97 ± 1.74	11.8 ± 2.03
6	Femoral	7.99 ± 1.9	8.26 ± 2.47	**7.1 ± 1.57**	7.42 ± 1.79	8.31 ± 1.64	8.32 ± 2.03
	Acetabular	8.23 ± 2.52	8.15 ± 2.58	**8.01 ± 2.4**	10.2 ± 3.53	11.3 ± 3.69	15.2 ± 3.91
	All Cartilage	8.1 ± 1.85	8.22 ± 2.06	**7.48 ± 1.52**	8.63 ± 2.32	9.68 ± 1.92	11.8 ± 2.14
8	Femoral	6.54 ± 2.22	**5.75 ± 1.55**	6.19 ± 1.61	9.25 ± 2.67	12.2 ± 3.45	8.17 ± 2.32
	Acetabular	7.67 ± 2.48	**7.22 ± 2.37**	8.2 ± 2.4	11.4 ± 3.26	11.8 ± 2.98	12.9 ± 3.52
	All Cartilage	6.97 ± 1.93	**6.33 ± 1.33**	6.98 ± 1.45	10.2 ± 2.72	12.0 ± 2.64	10.5 ± 2.3
10	Femoral	8.77 ± 3.12	**7.6 ± 1.46**	8.63 ± 4.69	8.38 ± 1.96	9.88 ± 2.35	8.1 ± 2.83
	Acetabular	9.26 ± 3.04	9.03 ± 2.49	**8.8 ± 2.92**	11.4 ± 3.5	11.1 ± 3.47	12.4 ± 3.37
	All Cartilage	8.99 ± 2.65	**8.12 ± 1.28**	8.7 ± 3.46	9.74 ± 2.24	10.5 ± 1.91	10.2 ± 2.4
12	Femoral	7.13 ± 1.78	7.92 ± 2.44	**6.49 ± 1.34**	8.34 ± 1.71	10.9 ± 2.92	8.69 ± 3.09
	Acetabular	8.64 ± 2.24	8.77 ± 2.5	**8.59 ± 2.29**	11.5 ± 3.72	12.1 ± 3.6	11.9 ± 3.37
	All Cartilage	7.75 ± 1.5	8.27 ± 2.19	**7.34 ± 1.38**	9.74 ± 2.23	11.5 ± 2.36	10.3 ± 2.52

Supplementary Information Table S9: T_2 quantification errors in hip cartilage compartments for 6 tested models. Performances of all methods in predicting T_2 maps in hip cartilage. NRMSEs are reported ±1 s.d., and the top performing model in each cartilage compartment at a given R is bolded (n=15). The no RNN pipeline version performs strongest at most R and cartilage compartments, and ROI-specific losses see stronger performance at most R and cartilage compartments than alternatively. In femoral cartilage, T_2 quantification errors are below clinically significant thresholds at nearly all tested R for the full model and no RNN pipelines. In acetabular cartilage, error rates were below clinically significant thresholds at R=2.

R	Tissue	Full Model	Reduced Parameters	No RNN	MANTIS	MANTIS-GAN	CS
2	Femoral	**0.773*****	0.765***	0.760***	0.717***	0.540***	0.399***
	Acetabular	**0.788*****	0.780***	0.753***	0.676***	0.481***	0.309***
	All Cartilage	**0.794*****	0.782***	0.770***	0.716***	0.514***	0.310***
3	Femoral	0.711***	**0.723*****	0.712***	0.587***	0.420***	0.414***
	Acetabular	0.660***	**0.709*****	0.663***	0.545***	0.315***	0.331***
	All Cartilage	0.705***	**0.726*****	0.703***	0.596***	0.372***	0.332***
4	Femoral	0.628***	0.635***	**0.641*****	0.528***	0.368***	0.408***
	Acetabular	0.620***	**0.656*****	0.616***	0.440***	0.294***	0.328***
	All Cartilage	0.646***	**0.665*****	0.648***	0.510***	0.333***	0.339***
6	Femoral	0.589***	**0.608*****	0.593***	0.422***	0.371***	0.428***
	Acetabular	0.551***	**0.558*****	0.521***	0.316	0.296***	0.292***
	All Cartilage	0.587***	**0.597*****	0.570***	0.382***	0.321***	0.334***
8	Femoral	**0.579*****	0.564***	0.555***	0.402***	0.331***	0.423***
	Acetabular	**0.576*****	0.579***	0.517***	0.229*	0.177**	0.323***
	All Cartilage	**0.598*****	0.588***	0.558***	0.334***	0.237***	0.347***
10	Femoral	0.523***	**0.528*****	0.511***	0.369***	0.333***	0.416***
	Acetabular	**0.542*****	0.482***	0.490***	0.177*	0.242***	0.308*
	All Cartilage	**0.558*****	0.534***	0.522***	0.279***	0.268***	0.335***
12	Femoral	0.521***	**0.563*****	0.508***	0.336***	0.299***	0.416***
	Acetabular	0.471***	**0.521*****	0.455***	0.192*	0.187**	0.333**
	All Cartilage	0.517***	**0.566*****	0.512***	0.280***	0.228***	0.349***

Supplementary Information Table S10: Correlation between predicted and ground truth T_2 in hip cartilage compartments for 6 tested models. Pearson's r between predicted and ground truth T_2 maps in hip cartilage with significances reported as follows: * $P < 0.05$, ** $P < 0.01$, *** $P < 0.001$ (n=15). The top performing model in each cartilage compartment at each R is shown in bold. The full model and reduced parameters pipelines generally show highest correlations between predicted and ground truth maps, but similar to the knee, networks with ROI-specific losses all show strong performance at high R.

R	Tissue	Full Model	Reduced Parameters	No RNN	MANTIS	MANTIS-GAN	CS
2	L1/L2	6.11 ± 1.3	6.17 ± 1.64	**5.36 ± 1.16**	6.91 ± 1.63	7.54 ± 2.19	10.0 ± 5.72
	L2/L3	9.08 ± 4.57	9.55 ± 5.41	**7.71 ± 5.98**	12.6 ± 11.1	13.0 ± 10.6	13.5 ± 6.28
	L3/L4	5.93 ± 1.1	5.82 ± 0.731	**3.99 ± 1.27**	7.93 ± 2.43	7.77 ± 2.56	10.1 ± 4.69
	L4/L5	5.86 ± 2.29	6.02 ± 2.22	**3.66 ± 0.817**	9.51 ± 3.23	9.71 ± 3.2	9.64 ± 3.82
	L5/S1	6.37 ± 2.1	7.77 ± 2.57	**4.18 ± 1.46**	7.48 ± 1.57	7.28 ± 2.04	6.31 ± 3.5
	All Discs	6.71 ± 1.7	6.86 ± 1.57	**4.86 ± 1.16**	8.78 ± 2.08	8.95 ± 1.91	10.1 ± 3.06
3	L1/L2	9.4 ± 1.83	8.16 ± 1.25	**7.37 ± 1.86**	9.59 ± 1.06	10.4 ± 2.51	10.9 ± 3.45
	L2/L3	12.6 ± 5.13	11.3 ± 6.64	**10.4 ± 7.42**	14.0 ± 7.2	15.4 ± 10.2	12.5 ± 5.32
	L3/L4	9.0 ± 2.76	7.96 ± 2.67	**6.14 ± 2.07**	10.8 ± 2.62	9.34 ± 2.1	9.75 ± 3.15
	L4/L5	8.57 ± 2.29	7.31 ± 1.84	**5.41 ± 0.985**	11.1 ± 1.86	12.1 ± 3.33	8.2 ± 1.33
	L5/S1	10.1 ± 2.22	10.7 ± 2.63	**5.9 ± 2.02**	8.9 ± 2.56	8.93 ± 2.32	5.82 ± 2.37
	All Discs	9.92 ± 2.39	8.76 ± 2.16	**7.13 ± 1.69**	11.0 ± 1.17	11.3 ± 1.74	9.48 ± 1.4
4	L1/L2	12.4 ± 3.58	10.3 ± 3.95	**9.21 ± 1.43**	11.3 ± 2.9	11.6 ± 4.09	13.2 ± 5.38
	L2/L3	12.1 ± 6.67	11.9 ± 7.16	**10.4 ± 7.1**	16.0 ± 8.03	18.9 ± 12.0	15.3 ± 5.64
	L3/L4	8.68 ± 3.86	8.42 ± 2.69	**6.14 ± 1.37**	11.1 ± 1.56	10.7 ± 3.07	9.66 ± 3.77
	L4/L5	9.64 ± 2.48	9.44 ± 2.74	**5.85 ± 1.11**	12.2 ± 2.82	13.2 ± 3.08	10.1 ± 2.94
	L5/S1	10.3 ± 2.44	9.86 ± 2.73	**6.49 ± 2.15**	9.55 ± 2.29	9.38 ± 1.96	6.78 ± 2.28
	All Discs	10.3 ± 3.02	9.73 ± 3.07	**7.42 ± 1.1**	12.1 ± 1.24	12.6 ± 1.35	11.3 ± 2.31
6	L1/L2	16.7 ± 14.0	13.2 ± 4.88	**11.9 ± 5.19**	14.0 ± 4.9	10.9 ± 2.36	14.4 ± 5.43
	L2/L3	15.7 ± 9.23	14.5 ± 8.95	**13.6 ± 9.98**	22.6 ± 12.1	17.7 ± 12.3	15.3 ± 7.14
	L3/L4	11.0 ± 3.69	11.5 ± 3.46	**9.0 ± 2.42**	13.6 ± 4.32	10.2 ± 1.65	11.3 ± 4.24
	L4/L5	10.5 ± 1.14	11.5 ± 4.11	**8.4 ± 2.1**	15.7 ± 5.61	12.2 ± 2.86	11.3 ± 2.61
	L5/S1	11.5 ± 1.73	12.5 ± 2.76	**8.82 ± 2.77**	12.5 ± 3.21	10.8 ± 3.11	8.19 ± 2.29
	All Discs	12.1 ± 3.58	12.2 ± 4.11	**10.3 ± 3.31**	15.6 ± 2.65	12.1 ± 1.9	12.0 ± 2.76
8	L1/L2	15.3 ± 3.94	15.9 ± 5.87	**13.6 ± 4.45**	12.8 ± 4.49	12.3 ± 3.96	13.3 ± 3.8
	L2/L3	17.2 ± 8.8	17.1 ± 8.64	**16.3 ± 8.99**	19.5 ± 9.27	20.0 ± 14.2	16.8 ± 6.38
	L3/L4	12.3 ± 4.85	11.7 ± 3.84	10.9 ± 3.22	11.6 ± 2.54	**10.4 ± 1.87**	13.5 ± 6.05
	L4/L5	12.3 ± 4.25	11.6 ± 2.18	**10.3 ± 2.79**	11.7 ± 2.63	12.4 ± 3.83	11.6 ± 2.44
	L5/S1	12.6 ± 3.94	10.5 ± 2.74	**9.01 ± 2.43**	10.8 ± 1.87	9.97 ± 3.12	9.18 ± 3.05
	All Discs	13.4 ± 3.89	13.0 ± 2.63	**12.0 ± 3.07**	13.2 ± 1.42	12.7 ± 1.7	12.8 ± 2.53
10	L1/L2	17.2 ± 3.6	18.2 ± 3.37	18.0 ± 2.72	15.2 ± 5.16	**12.9 ± 3.58**	16.4 ± 6.34
	L2/L3	19.7 ± 6.8	**17.9 ± 5.99**	18.1 ± 7.09	20.5 ± 10.9	19.5 ± 12.8	18.9 ± 8.12
	L3/L4	14.9 ± 4.38	14.0 ± 2.94	14.7 ± 3.62	11.8 ± 3.72	**12.3 ± 3.79**	15.5 ± 6.71
	L4/L5	14.1 ± 5.13	13.9 ± 4.83	12.8 ± 3.39	12.6 ± 1.79	**12.2 ± 1.84**	14.0 ± 3.9
	L5/S1	12.1 ± 5.09	12.3 ± 3.77	10.5 ± 4.38	11.6 ± 3.35	10.5 ± 2.69	**9.97 ± 2.36**
	All Discs	15.3 ± 3.22	14.8 ± 2.78	14.8 ± 2.26	13.8 ± 1.57	**13.2 ± 1.81**	15.0 ± 3.77
12	L1/L2	26.9 ± 10.1	25.5 ± 4.78	26.8 ± 12.0	**14.3 ± 4.15**	**14.3 ± 3.96**	33.0 ± 19.8
	L2/L3	23.2 ± 5.89	25.2 ± 3.31	23.3 ± 7.19	24.7 ± 18.7	**19.6 ± 12.2**	30.1 ± 11.3
	L3/L4	16.8 ± 4.11	24.5 ± 3.2	18.1 ± 3.88	14.1 ± 4.45	**13.2 ± 2.61**	26.1 ± 14.2
	L4/L5	15.3 ± 4.13	21.9 ± 5.59	15.3 ± 3.43	14.0 ± 5.81	**13.5 ± 5.25**	22.7 ± 11.0
	L5/S1	13.6 ± 4.66	17.9 ± 3.72	13.1 ± 4.29	9.84 ± 1.98	**10.5 ± 2.49**	11.3 ± 2.7
	All Discs	18.1 ± 1.95	23.1 ± 2.71	18.8 ± 2.76	14.8 ± 3.03	**14.1 ± 1.88**	24.8 ± 11.2

Supplementary Information Table S11: T_2 quantification errors in lumbar spine IVD levels for 6 tested models. Performances of all methods in predicting T_2 maps in lumbar spine IVDs. NRMSEs are reported ±1 s.d., and the top performing model in each cartilage compartment at a given R is bolded (n=5). Through R=8, the no RNN pipeline performs best in predicting T_2 maps, while at R=10, the MANTIS-GAN pipeline performs best. It's possible that, given the substantially smaller lumbar spine dataset from which DL models were trained, the more complicated loss functions of the

ROI-specific loss approaches make it difficult to train at ultrafast R, and collection of a larger dataset is required to see higher quality predictions. Regardless, all pipeline versions saw predictions with error rates below clinically significant thresholds aggregated across all discs for all tested R, except CS at R=12.

R	Tissue	Full Model	Reduced Parameters	No RNN	MANTIS	MANTIS-GAN	CS
2	L1/L2	0.849***	0.855***	**0.853*****	0.782***	0.782***	0.749***
	L2/L3	0.826***	0.830***	**0.832*****	0.750***	0.745***	0.751***
	L3/L4	0.861	0.869***	**0.886*****	0.764***	0.755***	0.830***
	L4/L5	0.859***	0.856***	**0.888*****	0.738***	0.739***	0.823***
	L5/S1	0.793***	0.771***	**0.832*****	0.696***	0.671***	0.712***
	All Discs	0.865***	0.866***	**0.884*****	0.784***	0.785***	0.802***
3	L1/L2	0.796***	**0.802*****	0.778***	0.668***	0.644***	0.703***
	L2/L3	**0.791*****	0.782***	0.779***	0.654***	0.658***	0.715***
	L3/L4	0.824***	0.809***	**0.830*****	0.673***	0.695***	0.759***
	L4/L5	0.841***	0.826***	**0.853*****	0.696***	0.676***	0.795***
	L5/S1	0.710***	0.682***	**0.763*****	0.584***	0.611***	0.741***
	All Discs	**0.836*****	0.823***	0.832***	0.717***	0.712***	0.777***
4	L1/L2	0.732***	**0.766*****	0.745***	0.623***	0.641***	0.579***
	L2/L3	0.749***	**0.756*****	0.751***	0.610***	0.596***	0.622***
	L3/L4	0.810***	0.814***	**0.826*****	0.652***	0.666***	0.747***
	L4/L5	0.818***	0.828***	**0.853*****	0.654***	0.618***	0.751***
	L5/S1	0.675***	0.694***	**0.730*****	0.557***	0.542***	0.672***
	All Discs	0.799***	0.813***	**0.819*****	0.680***	0.671***	0.723***
6	L1/L2	**0.722*****	0.717***	0.689***	0.626***	0.612***	0.653***
	L2/L3	**0.735*****	0.718***	0.724***	0.586***	0.584***	0.677***
	L3/L4	**0.762*****	0.745***	0.762***	0.608***	0.641***	0.708***
	L4/L5	0.794***	0.772***	**0.797*****	0.656***	0.626***	0.727***
	L5/S1	0.642***	0.618***	**0.661*****	0.462***	0.472***	0.620***
	All Discs	**0.776*****	0.764***	0.771***	0.660***	0.658***	0.728***
8	L1/L2	**0.659*****	0.613***	0.637***	0.583***	0.574***	0.599***
	L2/L3	**0.699*****	0.686***	0.696***	0.513***	0.544	0.652***
	L3/L4	**0.737*****	0.719***	0.734***	0.591***	0.644***	0.674***
	L4/L5	0.745***	0.731***	**0.752*****	0.613***	0.624***	0.725***
	L5/S1	0.606***	0.578***	**0.627*****	0.464***	0.501***	0.557***
	All Discs	**0.742*****	0.723***	**0.742*****	0.631***	0.645***	0.695***
10	L1/L2	0.586***	**0.610*****	0.514***	0.587***	0.557***	0.563***
	L2/L3	0.656***	**0.660*****	0.632***	0.557	0.518*	0.590***
	L3/L4	**0.681*****	**0.681*****	0.652***	0.636***	0.614***	0.644***
	L4/L5	**0.715*****	0.706***	0.701***	0.649***	0.614***	0.686***
	L5/S1	0.564***	**0.568*****	0.557***	0.426***	0.498***	0.502***
	All Discs	0.695***	**0.700*****	0.672***	0.647***	0.636***	0.648***
12	L1/L2	0.545***	0.202**	0.513***	**0.566*****	0.509***	0.453***
	L2/L3	**0.607*****	0.237***	0.572***	0.561***	0.513***	0.488***
	L3/L4	**0.652*****	0.273***	0.616***	0.586***	0.527***	0.542***
	L4/L5	**0.684*****	0.435***	0.668***	0.654***	0.585**	0.669***
	L5/S1	0.508***	0.233***	0.525***	**0.569*****	0.507***	0.436***
	All Discs	**0.664*****	0.320***	0.643***	0.651***	0.614***	0.586***

Supplementary Information Table S12: Correlation between predicted and ground truth T_2 in lumbar spine IVDs for 6 tested models. Pearson's r between predicted and ground truth T_2 maps lumbar spine IVDs with significances reported as follows: * $P < 0.05$, ** $P < 0.01$, *** $P < 0.001$ (n=5). The top performing model at each IVD level at each R is shown in bold. The full model and no RNN pipelines show highest correlations between predicted and ground truth maps, and all pipelines with ROI-specific losses performed well, apart from the reduced parameters pipeline at R=12. T_2 quantification performances of our methods were therefore strong across in IVDs across tested R.

			R						
	Fold	Loss Component	2	3	4	6	8	10	12
Knee	1	λ_{L_1}	1	1	1	1	1	1	1
		$\lambda_{L_{1,\phi}}$	123.5	144.5	145.6	120.8	144	110.8	78.9
		λ_{SSIM}	1.151	0.803	0.507	0.561	0.542	0.029	0.297
		$\lambda_{Feat.}$	0.101	0.499	0.447	0.132	0.128	0.138	0.131
	2	λ_{L_1}	1	1	1	1	1	1	1
		$\lambda_{L_{1,\phi}}$	117.5	100.2	124.3	120.8	61.8	64.7	76
		λ_{SSIM}	1.574	0.635	0.62	0.561	0.447	0.45	0.655
		$\lambda_{Feat.}$	0.433	0.423	0.395	0.132	0.451	0.367	0.134
	3	λ_{L_1}	1	1	1	1	1	1	1
		$\lambda_{L_{1,\phi}}$	144.4	100.2	145.6	137.9	118.4	134.9	106.9
		λ_{SSIM}	0.469	0.635	0.507	1.363	0.793	0.371	0.403
		$\lambda_{Feat.}$	0.119	0.423	0.447	0.429	0.389	0.115	0.035
Hip	1	λ_{L_1}	1	1	1	1	1	1	1
		$\lambda_{L_{1,\phi}}$	1.275	0.778	0.789	1.221	1.376	2.82	1.227
		λ_{SSIM}	0.27	1.7	1.609	1.727	1.253	0.948	0.961
		$\lambda_{Feat.}$	0.313	0.487	0.632	0.995	0.296	0.996	0.101
	2	λ_{L_1}	1	1	1	1	1	1	1
		$\lambda_{L_{1,\phi}}$	1.275	1.294	1.322	2.575	0.784	2.82	1.445
		λ_{SSIM}	0.27	0.728	1.215	1.749	1.27	0.948	0.917
		$\lambda_{Feat.}$	0.313	0.43	0.873	0.798	0.01	0.996	0.265
	3	λ_{L_1}	1	1	1	1	1	1	1
		$\lambda_{L_{1,\phi}}$	1.275	1.294	0.789	2.575	0.782	2.82	1.445
		λ_{SSIM}	0.27	0.728	1.609	1.749	1.576	0.948	0.917
		$\lambda_{Feat.}$	0.313	0.43	0.632	0.798	0.348	0.996	0.265
Lumbar Spine	1	λ_{L_1}	1	1	1	1	1	1	1
		$\lambda_{L_{1,\phi}}$	2.107	7.607	6.787	8.269	6.952	3.284	9.145
		λ_{SSIM}	40.336	69.261	95.355	70.068	19.915	72.269	88.492
		$\lambda_{Feat.}$	9.16	20.149	19.651	6.368	6.347	21.222	33.647
	2	λ_{L_1}	1	1	1	1	1	1	1
		$\lambda_{L_{1,\phi}}$	1.961	3.057	3.33	1.737	3.284	9.234	9.964
		λ_{SSIM}	73.233	87.205	67.15	94.58	72.269	85.043	62.233
		$\lambda_{Feat.}$	37.602	48.727	5.506	28.705	21.222	44.598	22.239
	3	λ_{L_1}	1	1	1	1	1	1	1
		$\lambda_{L_{1,\phi}}$	9.914	3.057	8.09	8.942	7.613	4.565	3.551
		λ_{SSIM}	49.478	87.205	41.3	67.49	61.799	69.246	72.122
		$\lambda_{Feat.}$	35.377	48.727	26.156	20.46	15.093	19.953	13.167

Supplementary Information Table S13: Optimized loss function weightings for best pipelines in each anatomy. Loss function weightings found to yield optimal pipeline performance through constrained hyperparameter searches in each anatomy, across each of the three folds. Across different folds at a given R in the same anatomy, optimized loss function weights generally, although not always, exhibited consistency with one another, indicating stability of training procedure.

			R						
**Tissue**	**Tissue Type**	**Fold**	**2**	**3**	**4**	**6**	**8**	**10**	**12**
Knee	Lateral Femoral Condyle	1	5.76 ± 2.63	7.37 ± 5.35	10.7 ± 10.1	10.9 ± 8.25	11.6 ± 9.1	13.2 ± 10.9	13.0 ± 7.39
		2	3.73 ± 1.27	5.99 ± 2.24	7.51 ± 4.79	8.93 ± 4.96	11.0 ± 4.8	9.67 ± 5.43	11.9 ± 5.55
		3	5.22 ± 1.73	6.76 ± 1.99	7.33 ± 2.8	7.4 ± 2.34	8.66 ± 3.54	9.52 ± 3.64	11.6 ± 3.59
	Lateral Tibial Condyle	1	6.02 ± 2.35	8.01 ± 3.83	9.2 ± 4.64	10.3 ± 4.75	10.4 ± 4.13	11.5 ± 5.28	13.3 ± 4.03
		2	5.98 ± 2.51	8.83 ± 3.17	12.1 ± 3.45	14.9 ± 3.69	13.7 ± 3.97	14.9 ± 3.77	15.7 ± 5.28
		3	8.42 ± 2.53	8.33 ± 2.73	12.1 ± 7.25	10.9 ± 4.61	10.9 ± 4.5	14.1 ± 4.5	14.8 ± 4.24
	Medial Femoral Condyle	1	5.31 ± 2.06	5.77 ± 2.19	7.16 ± 2.85	7.93 ± 3.36	9.5 ± 5.11	12.2 ± 5.9	12.1 ± 5.68
		2	3.34 ± 1.19	4.84 ± 2.22	6.12 ± 3.41	6.89 ± 3.98	7.78 ± 3.78	8.2 ± 4.84	11.0 ± 6.39
		3	4.18 ± 1.05	5.34 ± 1.32	6.09 ± 2.41	7.51 ± 2.57	7.64 ± 2.21	8.13 ± 2.18	10.0 ± 2.15
	Medial Tibial Condyle	1	8.1 ± 4.94	10.6 ± 8.22	11.3 ± 10.2	12.4 ± 8.32	12.9 ± 8.83	17.4 ± 14.3	15.0 ± 7.12
		2	4.5 ± 2.41	8.29 ± 4.0	10.3 ± 5.05	12.9 ± 5.7	10.1 ± 4.49	11.5 ± 4.53	11.7 ± 4.71
		3	8.51 ± 3.2	8.1 ± 3.32	13.5 ± 12.0	11.6 ± 4.74	12.3 ± 5.5	14.5 ± 4.17	15.2 ± 5.2
	Trochlear	1	7.19 ± 4.45	7.13 ± 4.43	7.4 ± 4.97	7.0 ± 4.39	8.49 ± 4.77	8.88 ± 6.27	8.6 ± 4.45
		2	4.67 ± 4.1	6.66 ± 3.86	8.47 ± 4.49	9.24 ± 4.35	13.9 ± 6.01	8.82 ± 4.28	12.8 ± 6.16
		3	5.11 ± 3.31	6.58 ± 3.59	5.67 ± 3.07	6.13 ± 2.6	7.83 ± 4.55	8.69 ± 4.59	7.72 ± 4.23
	Patellar	1	4.08 ± 1.24	5.03 ± 1.39	5.11 ± 1.26	5.69 ± 1.29	6.33 ± 1.24	6.31 ± 1.67	8.18 ± 1.95
		2	3.43 ± 2.44	7.4 ± 3.0	8.51 ± 2.82	11.3 ± 3.55	13.4 ± 4.01	8.98 ± 3.88	12.5 ± 5.65
		3	5.51 ± 2.9	6.65 ± 2.87	6.17 ± 2.68	7.32 ± 2.65	7.34 ± 3.2	7.69 ± 3.4	9.99 ± 3.84
	All Cartilage	1	5.52 ± 1.25	6.52 ± 2.17	7.54 ± 2.96	8.09 ± 2.65	8.94 ± 2.66	9.77 ± 3.44	10.7 ± 2.32
		2	4.11 ± 2.12	6.8 ± 2.38	8.45 ± 2.86	10.4 ± 3.12	12.6 ± 3.47	9.76 ± 3.47	12.7 ± 4.52
		3	5.82 ± 1.98	6.88 ± 2.17	7.33 ± 2.35	7.99 ± 2.2	8.7 ± 2.71	9.8 ± 2.61	11.1 ± 2.5
Hip	Femoral	1	3.69 ± 1.0	6.14 ± 1.61	5.66 ± 1.01	7.99 ± 1.9	6.54 ± 2.22	8.77 ± 3.12	7.13 ± 1.78
		2	4.0 ± 1.69	4.93 ± 1.68	7.86 ± 2.86	6.66 ± 2.51	8.38 ± 3.04	6.86 ± 2.66	6.79 ± 3.02
		3	3.36 ± 0.86	4.17 ± 1.05	5.14 ± 1.02	5.89 ± 1.72	7.36 ± 1.56	6.6 ± 2.24	6.42 ± 1.63
	Acetabular	1	4.4 ± 1.46	7.16 ± 2.31	7.0 ± 1.76	8.23 ± 2.52	7.67 ± 2.48	9.26 ± 3.04	8.64 ± 2.24
		2	5.01 ± 3.84	5.9 ± 4.09	8.65 ± 5.94	8.0 ± 5.15	9.42 ± 5.42	8.54 ± 5.72	9.38 ± 5.54
		3	3.18 ± 0.629	4.41 ± 1.11	5.52 ± 1.18	6.73 ± 1.99	8.3 ± 2.34	6.97 ± 2.29	7.06 ± 2.1
	All Cartilage	1	3.97 ± 1.03	6.53 ± 1.63	6.15 ± 1.01	8.1 ± 1.85	6.97 ± 1.93	8.99 ± 2.65	7.75 ± 1.5
		2	4.42 ± 2.44	5.33 ± 2.5	8.23 ± 3.81	7.21 ± 3.46	8.84 ± 3.71	7.52 ± 3.72	7.84 ± 3.94
		3	3.31 ± 0.661	4.29 ± 0.865	5.29 ± 0.889	6.28 ± 1.42	7.79 ± 1.32	6.78 ± 1.98	6.73 ± 1.35
Lumbar Spine	L1/L2	1	6.11 ± 1.3	9.4 ± 1.83	12.4 ± 3.58	16.7 ± 14.0	15.3 ± 3.94	17.2 ± 3.6	26.9 ± 10.1
		2	8.93 ± 4.5	16.0 ± 7.29	16.7 ± 8.83	25.4 ± 13.0	25.5 ± 14.3	20.1 ± 9.02	20.7 ± 9.87
		3	8.88 ± 5.43	8.84 ± 2.08	16.4 ± 3.98	19.1 ± 9.7	20.2 ± 12.7	20.0 ± 8.87	27.3 ± 9.65
	L2/L3	1	9.08 ± 4.57	12.6 ± 5.13	12.1 ± 6.67	15.7 ± 9.23	17.2 ± 8.8	19.7 ± 6.8	23.2 ± 5.89
		2	9.09 ± 4.82	15.6 ± 10.0	17.6 ± 11.7	33.4 ± 24.7	34.7 ± 23.8	27.8 ± 14.0	24.4 ± 15.8
		3	8.82 ± 4.73	7.74 ± 1.56	16.6 ± 10.2	16.8 ± 10.8	20.6 ± 12.2	20.1 ± 13.3	26.6 ± 14.4
	L3/L4	1	5.93 ± 1.1	9.0 ± 2.76	8.68 ± 3.86	11.0 ± 3.69	12.3 ± 4.85	14.9 ± 4.38	16.8 ± 4.11
		2	6.45 ± 3.06	15.1 ± 9.9	10.3 ± 5.24	27.6 ± 19.1	25.7 ± 16.7	26.1 ± 11.5	23.3 ± 14.0
		3	6.52 ± 2.02	7.85 ± 1.13	13.6 ± 4.0	13.3 ± 5.22	17.1 ± 8.4	23.4 ± 13.4	35.3 ± 22.0
	L4/L5	1	5.86 ± 2.29	8.57 ± 2.29	9.64 ± 2.48	10.5 ± 1.14	12.3 ± 4.25	14.1 ± 5.13	15.3 ± 4.13
		2	9.08 ± 4.79	16.5 ± 8.71	16.4 ± 8.58	27.2 ± 15.4	24.1 ± 12.6	27.4 ± 12.8	24.7 ± 12.0
		3	7.65 ± 3.82	8.68 ± 1.01	14.8 ± 7.36	16.0 ± 8.91	17.5 ± 8.36	20.4 ± 9.49	29.7 ± 13.9
	L5/S1	1	6.37 ± 2.1	10.1 ± 2.22	10.3 ± 2.44	11.5 ± 1.73	12.6 ± 3.94	12.1 ± 5.09	13.6 ± 4.66
		2	10.2 ± 7.62	17.8 ± 14.2	23.4 ± 17.0	20.8 ± 12.7	21.8 ± 12.6	30.8 ± 13.7	23.0 ± 13.5
		3	5.17 ± 1.3	9.42 ± 2.17	7.96 ± 1.16	9.01 ± 1.46	8.99 ± 0.531	12.4 ± 1.59	13.5 ± 1.23
	All Discs	1	6.71 ± 1.7	9.92 ± 2.39	10.3 ± 3.02	12.1 ± 3.58	13.4 ± 3.89	15.3 ± 3.22	18.1 ± 1.95
		2	8.45 ± 3.74	16.3 ± 8.33	14.8 ± 7.18	28.3 ± 16.5	26.5 ± 14.5	26.5 ± 11.5	23.5 ± 11.4
		3	7.65 ± 3.58	8.66 ± 0.601	13.8 ± 5.74	14.4 ± 7.25	16.2 ± 8.06	19.2 ± 10.1	26.3 ± 13.4

Supplementary Information Table S14: T_2 quantification error rates across 3 splits in tissues of interest. NRMSEs reported reported ±1 s.d. between ground truth and predicted T_2 maps in across cartilage compartments and IVD levels in 3 data splits (knee: n=16, n=9, n=16 for folds 1-3, respectively; hip: n=15 for each of folds 1-3; lumbar spine: n=5, n=4, n=4

for folds 1-3, respectively). Particularly for knee and hip pipelines, performance is consistent across data splits in cartilage compartments and overall at all tested R. In lumbar spine, performance showed increased variability compared to knee and hip pipelines, but mean T_2 quantification errors were all within a standard deviation of one another. Relatively small lumbar spine dataset size relative to knee and hip dataset sizes are likely responsible for considerably wider confidence intervals and increased variance in performance for lumbar spine.

			R						
**Tissue**	**Tissue Type**	**Fold**	**2**	**3**	**4**	**6**	**8**	**10**	**12**
Knee	Lateral Femoral Condyle	1	0.712***	0.655***	0.528***	0.475***	0.473***	0.399	0.359*
		2	0.769***	0.666***	0.604***	0.535***	0.454***	0.458***	0.380***
		3	0.770***	0.695***	0.633***	0.588***	0.528**	0.516***	0.334***
	Lateral Tibial Condyle	1	0.801***	0.715***	0.682***	0.600**	0.601***	0.548*	0.470***
		2	0.821***	0.715***	0.591***	0.537***	0.536***	0.434***	0.451***
		3	0.760***	0.728***	0.658***	0.622***	0.591***	0.478	0.428***
	Medial Femoral Condyle	1	0.759***	0.723***	0.666***	0.606***	0.590***	0.509***	0.444***
		2	0.757***	0.658***	0.580***	0.528***	0.484***	0.443***	0.438***
		3	0.771***	0.683***	0.628***	0.476***	0.499***	0.462***	0.305***
	Medial Tibial Condyle	1	0.721***	0.655*	0.600*	0.530**	0.502*	0.427***	0.339*
		2	0.784***	0.620***	0.495***	0.360*	0.461***	0.308***	0.321
		3	0.656***	0.637***	0.553*	0.484*	0.407*	0.247	0.153
	Trochlear	1	0.780***	0.711***	0.710***	0.728***	0.691***	0.650***	0.656***
		2	0.824***	0.773***	0.728***	0.717***	0.664***	0.660***	0.657***
		3	0.817***	0.762***	0.715***	0.717***	0.701***	0.661***	0.678***
	Patellar	1	0.671***	0.618***	0.627***	0.557***	0.554***	0.537***	0.434***
		2	0.839***	0.738***	0.683***	0.609***	0.557***	0.578***	0.504***
		3	0.805***	0.771***	0.733***	0.613***	0.659***	0.590***	0.477***
	All Cartilage	1	0.748***	0.695***	0.651***	0.612***	0.585***	0.555***	0.491***
		2	0.812***	0.709***	0.633***	0.569***	0.532***	0.519	0.485***
		3	0.775***	0.728***	0.666***	0.597***	0.583***	0.521***	0.450***
Hip	Femoral	1	0.773***	0.711***	0.628***	0.589***	0.579***	0.523***	0.521***
		2	0.755***	0.686***	0.611***	0.585***	0.564***	0.518***	0.554***
		3	0.802***	0.738***	0.667***	0.623***	0.537***	0.581***	0.593***
	Acetabular	1	0.788***	0.660***	0.620***	0.551***	0.576***	0.542***	0.471***
		2	0.792***	0.722***	0.613***	0.586***	0.541***	0.553***	0.481***
		3	0.821***	0.744***	0.677***	0.566***	0.468***	0.552***	0.543***
	All Cartilage	1	0.794***	0.705***	0.646***	0.587***	0.598***	0.558***	0.517***
		2	0.782***	0.714***	0.624***	0.594***	0.564***	0.554***	0.539***
		3	0.818***	0.753***	0.687***	0.616***	0.519***	0.586***	0.589***
Lumbar Spine	L1/L2	1	0.849***	0.796***	0.732***	0.722***	0.659***	0.586***	0.545***
		2	0.793***	0.704***	0.715***	0.572***	0.492***	0.323***	0.462***
		3	0.812***	0.741***	0.722***	0.707***	0.696***	0.579***	0.521***
	L2/L3	1	0.826***	0.791***	0.749***	0.735***	0.699***	0.656***	0.607***
		2	0.869***	0.824***	0.824***	0.701***	0.641***	0.375***	0.584***
		3	0.852***	0.791***	0.776***	0.779***	0.728***	0.651***	0.569***
	L3/L4	1	0.861***	0.824***	0.810***	0.762***	0.737***	0.681***	0.652***
		2	0.896***	0.850***	0.855***	0.740***	0.687***	0.291***	0.604***
		3	0.861***	0.783***	0.755***	0.756***	0.722***	0.628***	0.514***
	L4/L5	1	0.859***	0.841***	0.818***	0.794***	0.745***	0.715***	0.684***
		2	0.835***	0.757***	0.709***	0.642***	0.607***	0.357***	0.539***
		3	0.823***	0.738***	0.709***	0.717***	0.671***	0.578***	0.540***
	L5/S1	1	0.793***	0.710***	0.675***	0.642***	0.606***	0.564***	0.508***
		2	0.769***	0.657***	0.649***	0.546***	0.490***	0.150	0.469***
		3	0.878***	0.792***	0.737***	0.747***	0.707***	0.602***	0.563***
	All Discs	1	0.865***	0.836***	0.799***	0.776***	0.742***	0.695***	0.664***
		2	0.855***	0.798***	0.788***	0.682***	0.622***	0.281***	0.565***
		3	0.859***	0.781***	0.767***	0.774	0.724***	0.647***	0.576***

Supplementary Information Table S15: Correlations between predicted and ground truth T_2 maps across 3 splits in tissues of interest. Pearson's r between predicted and ground truth T_2 maps in tissues of interest for knee, hip and lumbar

spine pipelines, with significances reported as follows: * $P < 0.05$, ** $P < 0.01$, *** $P < 0.001$ (knee: n=16, n=9, n=16 for folds 1-3, respectively; hip: n=15 for each of folds 1-3; lumbar spine: n=5, n=4, n=4 for folds 1-3, respectively). Performance is reported across each of the 3 data splits. With few exceptions across some IVD levels for some R, deviations in Pearson's r were relatively small across splits for the same tissue of interest at a given R, indicating stability of pipelines to datasets used.

	R	Fold	GLCM Texture Metric				
			**Contrast**	**Dissimilarity**	**Homogeneity**	**ASM**	**Energy**
**Knee**	**2**	1	0.307 ± 0.18**	0.638 ± 0.12***	0.734 ± 0.09***	0.966 ± 0.015***	0.954 ± 0.02***
		2	0.344 ± 0.24**	0.493 ± 0.2***	0.673 ± 0.15***	0.908 ± 0.05***	0.902 ± 0.05***
		3	0.261 ± 0.18**	0.444 ± 0.16***	0.579 ± 0.13***	0.906 ± 0.04***	0.909 ± 0.035***
	**3**	1	0.153 ± 0.2	0.521 ± 0.15***	0.735 ± 0.09***	0.962 ± 0.015***	0.95 ± 0.02***
		2	0.0972 ± 0.26	0.157 ± 0.26	0.299 ± 0.24*	0.874 ± 0.07***	0.875 ± 0.07***
		3	0.256 ± 0.18**	0.41 ± 0.17***	0.474 ± 0.16***	0.885 ± 0.045***	0.895 ± 0.04***
	**4**	1	0.11 ± 0.2	0.387 ± 0.17***	0.61 ± 0.12***	0.973 ± 0.01***	0.95 ± 0.02***
		2	0.0554 ± 0.26	-5.23e-06 ± 0.27	0.0101 ± 0.26	0.838 ± 0.08***	0.819 ± 0.09***
		3	0.179 ± 0.2*	0.448 ± 0.16***	0.625 ± 0.12***	0.908 ± 0.04***	0.908 ± 0.035***
	**6**	1	0.0667 ± 0.2	0.22 ± 0.19*	0.382 ± 0.17***	0.97 ± 0.015***	0.94 ± 0.025***
		2	0.0219 ± 0.27	-0.0819 ± 0.26	-0.125 ± 0.26	0.819 ± 0.09***	0.808 ± 0.095***
		3	0.304 ± 0.18**	0.458 ± 0.16***	0.525 ± 0.15***	0.879 ± 0.05***	0.885 ± 0.045***
	**8**	1	0.061 ± 0.2	0.111 ± 0.2	0.0615 ± 0.2	0.952 ± 0.02***	0.9 ± 0.04***
		2	-0.00262 ± 0.26	-0.133 ± 0.26	-0.187 ± 0.26	0.825 ± 0.085***	0.799 ± 0.1***
		3	0.0692 ± 0.2	0.0851 ± 0.2	0.15 ± 0.2	0.835 ± 0.065***	0.829 ± 0.065***
	**10**	1	0.0594 ± 0.2	0.218 ± 0.19*	0.307 ± 0.18**	0.961 ± 0.015***	0.928 ± 0.03***
		2	0.0279 ± 0.26	-0.0231 ± 0.26	-0.0865 ± 0.26	0.831 ± 0.085***	0.808 ± 0.095***
		3	-0.0143 ± 0.2	-0.0469 ± 0.2	0.0504 ± 0.2	0.769 ± 0.085***	0.764 ± 0.085***
	**12**	1	0.0032 ± 0.2	-0.066 ± 0.2	-0.178 ± 0.19	0.927 ± 0.03***	0.861 ± 0.055***
		2	-0.0137 ± 0.26	-0.186 ± 0.26	-0.327 ± 0.24	0.807 ± 0.095***	0.772 ± 0.11***
		3	-0.00712 ± 0.2	-0.133 ± 0.2	-0.166 ± 0.19	0.756 ± 0.09***	0.742 ± 0.09***
**Hip**	**2**	1	0.312 ± 0.34*	0.633 ± 0.23***	0.837 ± 0.12***	0.945 ± 0.04***	0.957 ± 0.035***
		2	0.116 ± 0.35	0.345 ± 0.32*	0.72 ± 0.18***	0.902 ± 0.07***	0.915 ± 0.065***
		3	0.274 ± 0.33	0.476 ± 0.28**	0.698 ± 0.19***	0.884 ± 0.085***	0.889 ± 0.085***
	**3**	1	0.369 ± 0.32*	0.671 ± 0.21***	0.816 ± 0.14***	0.976 ± 0.02***	0.98 ± 0.015***
		2	0.146 ± 0.35	0.415 ± 0.3*	0.836 ± 0.12***	0.923 ± 0.06***	0.917 ± 0.065***
		3	0.285 ± 0.33	0.504 ± 0.28**	0.721 ± 0.18***	0.942 ± 0.045***	0.937 ± 0.05***
	**4**	1	0.328 ± 0.33*	0.597 ± 0.25***	0.801 ± 0.15***	0.957 ± 0.035***	0.954 ± 0.04***
		2	0.0992 ± 0.36	0.294 ± 0.33	0.677 ± 0.2***	0.913 ± 0.065***	0.918 ± 0.065***
		3	0.423 ± 0.3**	0.649 ± 0.22***	0.824 ± 0.12***	0.914 ± 0.065***	0.913 ± 0.065***
	**6**	1	0.235 ± 0.35	0.475 ± 0.3**	0.645 ± 0.23***	0.939 ± 0.05***	0.941 ± 0.045***
		2	0.124 ± 0.35	0.386 ± 0.3*	0.791 ± 0.14***	0.907 ± 0.07***	0.902 ± 0.075***
		3	0.238 ± 0.34	0.464 ± 0.28**	0.731 ± 0.18***	0.892 ± 0.08***	0.893 ± 0.08***
	**8**	1	0.199 ± 0.36	0.487 ± 0.28**	0.823 ± 0.13***	0.923 ± 0.06***	0.933 ± 0.055***
		2	0.128 ± 0.35	0.375 ± 0.31*	0.764 ± 0.16***	0.848 ± 0.11***	0.82 ± 0.12***
		3	0.286 ± 0.33	0.521 ± 0.27**	0.772 ± 0.16***	0.839 ± 0.12***	0.844 ± 0.11***
	**10**	1	0.127 ± 0.36	0.308 ± 0.34	0.48 ± 0.29**	0.862 ± 0.11***	0.855 ± 0.11***
		2	0.0664 ± 0.36	0.209 ± 0.34	0.438 ± 0.3**	0.809 ± 0.13***	0.799 ± 0.14***
		3	0.135 ± 0.35	0.27 ± 0.33	0.473 ± 0.28**	0.695 ± 0.19***	0.73 ± 0.18***
	**12**	1	0.198 ± 0.36	0.38 ± 0.32*	0.523 ± 0.28**	0.927 ± 0.06***	0.914 ± 0.07***
		2	0.12 ± 0.36	0.376 ± 0.31*	0.708 ± 0.19***	0.818 ± 0.13***	0.8 ± 0.14***
		3	0.142 ± 0.34	0.317 ± 0.32*	0.57 ± 0.25***	0.795 ± 0.15***	0.813 ± 0.14***
**Lumbar Spine**	**2**	1	0.557 ± 0.7	0.695 ± 0.62	0.744 ± 0.57*	0.892 ± 0.35**	0.923 ± 0.27**
		2	0.449 ± 0.83	0.589 ± 0.78	0.788 ± 0.64	0.859 ± 0.54*	0.903 ± 0.44*
		3	-0.233 ± 0.86	-0.38 ± 0.84	0.376 ± 0.84	0.969 ± 0.2**	0.996 ± 0.03***
	**3**	1	0.499 ± 0.73	0.615 ± 0.67	0.644 ± 0.66	0.819 ± 0.48*	0.872 ± 0.39*
		2	0.419 ± 0.84	0.659 ± 0.74	0.616 ± 0.77	0.414 ± 0.84	0.532 ± 0.8

		3	-0.163 ± 0.87	-0.195 ± 0.87	0.391 ± 0.84	0.991 ± 0.065***	0.987 ± 0.09***
	**4**	1	0.236 ± 0.8	0.421 ± 0.76	0.497 ± 0.73	0.67 ± 0.64	0.775 ± 0.54*
		2	0.291 ± 0.86	0.515 ± 0.81	0.67 ± 0.74	0.551 ± 0.8	0.653 ± 0.75
		3	-0.175 ± 0.87	-0.186 ± 0.87	0.346 ± 0.85	0.942 ± 0.32**	0.954 ± 0.26**
	**6**	1	0.341 ± 0.78	0.428 ± 0.76	0.262 ± 0.8	0.566 ± 0.7	0.67 ± 0.64
		2	0.109 ± 0.88	0.356 ± 0.85	0.417 ± 0.84	0.347 ± 0.85	0.438 ± 0.84
		3	-0.189 ± 0.88	-0.219 ± 0.87	0.159 ± 0.88	0.936 ± 0.34**	0.958 ± 0.25**
	**8**	1	0.0633 ± 0.81	0.152 ± 0.8	0.276 ± 0.79	0.685 ± 0.62	0.728 ± 0.58
		2	-0.169 ± 0.87	0.043 ± 0.88	0.319 ± 0.86	0.67 ± 0.74	0.681 ± 0.74
		3	-0.061 ± 0.88	0.00747 ± 0.88	0.376 ± 0.84	0.938 ± 0.33**	0.958 ± 0.24**
	**10**	1	-0.0393 ± 0.81	-0.0631 ± 0.81	-0.0699 ± 0.81	0.403 ± 0.76	0.479 ± 0.74
		2	-0.393 ± 0.84	-0.361 ± 0.85	-0.194 ± 0.88	0.473 ± 0.82	0.496 ± 0.82
		3	-0.0191 ± 0.88	0.13 ± 0.88	0.434 ± 0.84	0.925 ± 0.37*	0.95 ± 0.29**
	**12**	1	-0.0697 ± 0.81	-0.156 ± 0.8	-0.424 ± 0.76	0.16 ± 0.8	0.198 ± 0.8
		2	0.104 ± 0.88	0.377 ± 0.84	0.435 ± 0.84	0.505 ± 0.82	0.548 ± 0.8
		3	-0.0992 ± 0.88	-0.000956 ± 0.88	0.249 ± 0.86	0.968 ± 0.2**	0.958 ± 0.25**

Supplementary Information Table S16: Texture retention performance of knee, hip and lumbar spine pipelines across 3 splits in tissues of interest. Intraclass correlation coefficients (ICCs) of Gray Level Co-Occurrence Matrix (GLCM)-based metrics for knee, hip and lumbar spine pipelines across 3 data splits. Significance of ICCs is reported as follows: * $P < 0.05$, ** $P < 0.01$, *** $P < 0.001$ (knee: n=16, n=9, n=16 for folds 1-3, respectively; hip: n=15 for each of folds 1-3; lumbar spine: n=5, n=4, n=4 for folds 1-3, respectively). Deviation in ICCs for texture metrics is minimal in the hip and knee pipelines for all cartilage compartments at all tested R. In the lumbar spine pipeline, more deviation existed in texture metrics, although due to small test set sizes (n=4), confidence intervals for ICCs are very wide, so at least some of the differences in texture retention performance can be attributed to this.
